# Big data approaches to bovine bioacoustics: a FAIR-compliant dataset and scalable ML framework for precision livestock welfare

**DOI:** 10.3389/fdata.2025.1723155

**Published:** 2026-01-16

**Authors:** Mayuri Kate, Suresh Neethirajan

**Affiliations:** 1 Faculty of Computer Science, Dalhousie University, Halifax, NS, Canada; 2 Faculty of Agriculture, Dalhousie University, Bible Hill, NS, Canada

**Keywords:** acoustic feature extraction, animal welfare monitoring, bioacoustics, bovine vocalizations, FAIR data principles, machine learning in agriculture, multimodal dataset, precision livestock farming

## Abstract

The convergence of IoT sensing, edge computing, and machine learning is revolutionizing precision livestock farming. Yet bioacoustic data streams remain underexploited due to computational-complexity and ecological-validity challenges. We present one of the most comprehensive bovine vocalization datasets to date-569 expertly curated clips spanning 48 behavioral classes, recorded across three commercial dairy farms using multi-microphone arrays and expanded to 2,900 samples through domain-informed data augmentation. This FAIR-compliant resource addresses key Big Data challenges: volume (90 h of raw recordings, 65.6 GB), variety (multi-farm, multi-zone acoustic environments), velocity (real-time processing requirements), and veracity (noise-robust feature-extraction pipelines). A modular data-processing workflow combines denoising implemented both in iZotope RX 11 for quality control and an equivalent open-source Python pipeline using noisereduce, multi-modal synchronization (audio-video alignment), and standardized feature engineering (24 acoustic descriptors via Praat, librosa, and openSMILE) to enable scalable welfare monitoring. Preliminary machine-learning benchmarks reveal distinct class-wise acoustic signatures across estrus detection, distress classification, and maternal-communication recognition. The dataset's ecological realism-embracing authentic barn acoustics rather than controlled conditions-ensures deployment-ready model development. This work establishes the foundation for animal-centered AI, where bioacoustic streams enable continuous, non-invasive welfare assessment at industrial scale. By releasing a Zenodo-hosted, FAIR-compliant dataset (restricted access) and an open-source preprocessing pipeline on GitHub, together with comprehensive metadata schemas, we advance reproducible research at the intersection of Big Data analytics, sustainable agriculture, and precision livestock management. The framework directly supports UN SDG 9, demonstrating how data science can transform traditional farming into intelligent, welfare-optimized production systems capable of meeting global food demands while maintaining ethical animal-care standards.

## 1 Introduction

The exponential growth of agricultural data—projected to surpass 5.1 exabytes by 2025—positions precision livestock farming at the intersection of IoT sensing, edge computing, and machine learning analytics (Scoop Market Research, 2025; Precision Business Insights, 2024). Within this evolving digital ecosystem, bioacoustic data streams stand out as a particularly complex and information-rich modality. These continuous, high-frequency temporal signals demand specialized preprocessing pipelines, robust feature engineering, and scalable analysis frameworks to unlock actionable insights. Building on this context, the rapid growth of digital agriculture has further highlighted the transformative potential of big data and machine learning in reshaping livestock farming into a more sustainable, welfare-centered, and efficient sector. Among various sensing modalities, bioacoustics has emerged as a powerful yet underutilized channel of information, offering non-invasive insights into animal health, behavior, and emotional state. In particular, cattle vocalizations carry rich indicators of social interaction, estrus, maternal care, hunger, stress, and pain, positioning them as promising biomarkers for welfare monitoring and automated farm management systems. Harnessing these signals, however, requires curated datasets that faithfully capture the acoustic, behavioral, and environmental realities of commercial farming contexts.

The lack of large, annotated datasets remains one of the most significant bottlenecks in bovine bioacoustics research (Kate and Neethirajan, 2025). Traditional acoustic analysis methods involving manual spectrogram generation and feature extraction are informative but not scalable to the data volumes required for robust AI training, underscoring the critical need for comprehensive, FAIR (Findable, Accessible, Interoperable, Reusable)-compliant datasets that capture ecological validity while supporting big data analytics. Importantly, FAIR principles do not require that all data be fully open; rather, they emphasize transparent, machine-readable access conditions that can accommodate justified restrictions such as farm confidentiality (Martorana et al., 2022; Karakoltzidis et al., 2024).

Despite increasing interest, existing bovine vocalization corpora remain limited in scale, scope, and reproducibility. Most prior datasets have been collected from small cohorts under controlled or homogeneous conditions, focusing primarily on a narrow set of call types such as estrus calls or distress vocalizations. For instance, the “BovineTalk” dataset reported over a thousand vocalizations but from only 20 cows in isolation, thereby excluding environmental noise and behavioral diversity. Similarly, physiological studies linking calls to cortisol or estrus relied on restricted conditions, limiting generalizability to commercial barns. These constraints hinder the development of machine learning models that can generalize across diverse farm environments, rare behaviors, and variable acoustic conditions. Furthermore, multimodal integration of audio with video or ethological annotations is rarely implemented, restricting opportunities to contextualize vocalizations with corresponding behaviors.

In addition to limited behavioral coverage, many existing datasets deliberately exclude background noise to ensure clean acoustic signals. While this simplifies analysis, it reduces ecological validity, as commercial dairy barns are acoustically complex environments containing mechanical noise, overlapping calls, and human activity. Models trained on clean laboratory recordings often fail when deployed in real-world farms, where vocal signals are embedded within heterogeneous soundscapes. There is therefore an urgent need for datasets that reflect the acoustic reality of farming environments, balancing signal clarity with ecological authenticity.

To address these gaps, we introduce a novel bovine vocalization dataset that combines scale, behavioral diversity, and ecological realism with rigorous annotation and metadata standards. The corpus comprises 569 curated clips spanning 48 behavioral classes, recorded across three commercial dairy farms in Atlantic Canada using a multi-microphone, multimodal design. By capturing audio simultaneously from multiple barn zones—feeding alleys, drinking troughs, milking parlors, and resting pens–and pairing these recordings with video observations and detailed ethological notes, the dataset provides a comprehensive representation of the acoustic and behavioral ecology of dairy cattle. Unlike earlier collections that prioritized controlled conditions, this resource embraces the complexity of barn environments, including background machinery, overlapping calls, and routine human activity, thereby enhancing its value for developing robust, field-ready analytical models.

A key contribution of this dataset lies in its ethology-driven annotation scheme, which organizes vocalizations into nine main categories and 48 sub-types covering maternal, social, reproductive, feeding, drinking, handling, distress, environmental, and non-vocal events. Each clip is annotated with behavioral context, emotional valence, and confidence scores, enabling analyses that extend beyond acoustics to questions of welfare, motivation, and social interaction. This structure aligns with contemporary animal welfare frameworks that emphasize emotional valence and arousal, while also providing machine-readable descriptors suitable for computational modeling. Equally important is the dataset's adherence to FAIR principles. Metadata tables document recording context, equipment, clip features, and preprocessing parameters in a transparent and reproducible manner. The inclusion of acoustic features extracted with standardized pipelines (Praat, librosa, openSMILE) ensures interoperability with other livestock bioacoustic resources and facilitates downstream applications ranging from supervised classification to exploratory behavioral analysis.

Together, these elements establish this corpus as the most comprehensive and ecologically valid dataset of bovine vocalizations to date. It provides not only a foundation for advancing machine learning approaches to livestock sound analysis, but also a benchmark resource for researchers in animal behavior, welfare science, and precision livestock management. To support reproducible reuse, the curated clips and associated metadata are deposited under restricted access in a Zenodo repository with a persistent DOI, while the full preprocessing and feature extraction pipeline is released as open-source code on GitHub.

In addition to its methodological and scientific contributions, this dataset holds direct significance for the emerging field of precision livestock farming. By enabling the detection and interpretation of vocal cues linked to health, reproduction, and welfare, it opens pathways for non-invasive monitoring systems that can assist farmers in real time. Early detection of estrus, distress, or discomfort through automated vocal analysis could enhance reproductive management, reduce disease risks, and improve overall herd wellbeing. Beyond cattle, the dataset also contributes to the broader movement in animal-centered AI, where bioacoustic data are increasingly leveraged to give “digital voices” to non-human species. Recent work on AI-assisted behavioral monitoring in sheep and goats, for example, has demonstrated how vocal and behavioral traits can be mapped to welfare-relevant states using machine learning (Emsen et al., 2025). Our bovine vocalization dataset extends this line of work to dairy cattle, providing a complementary resource within a growing ecosystem of AI tools that integrate vocalization analysis with computer vision and wearable sensor data across ruminant species.

The remainder of this paper is structured as follows. Section 2 presents the novelty of the dataset, situating it in relation to previous studies. Section 3 describes the data collection protocols, including recording sites, equipment, and multimodal capture methods. Section 4 outlines the preprocessing pipeline, covering noise profiling, filtering, denoising, segmentation, and annotation. Section 5 details dataset creation, including feature extraction, biological interpretation, metadata design, and preliminary analyses. Finally, Section 6 presents the discussion of significance and limitations, and Section 7 concludes the paper highlighting its potential as a benchmark for both animal welfare science and big data applications in agriculture. Building on these motivations, the next section outlines the novelty of our dataset in relation to existing bovine vocalization corpora.

## 2 Dataset novelty

### 2.1 Scale and diversity of recordings

This work presents one of the most comprehensive bovine vocalization datasets to date. The corpus comprises 569 clips covering 48 behavioral labels (classes) and has a mean clip duration of ~ 21 s (median ~ 13.8 s; range 2.8–445 s). Analysis shows a long tailed distribution: the largest classes, Estrus_Call (117 clips) and Feed_Anticipation_Call (113 clips), account for 40% of the data, whereas many categories contain fewer than ten samples, reflecting the rare and spontaneous nature of some behaviors. Clip durations are short enough to facilitate fine grained acoustic analysis yet long enough to capture the full vocalization plus context. A detailed breakdown of the dataset composition is provided in Supplementary Table S1, which reports clip counts and total duration by main category, subcategory, farm, barn zone, and microphone. The underlying metadata and acoustic feature tables are distributed with the Zenodo record described in the Data Availability Statement, enabling other researchers to subset the corpus by behavioral class, recording context, or equipment configuration for targeted analyses.

Most published bovine call datasets are smaller both in scale and scope. For example, the “BovineTalk” study isolated 20 multiparous cows for 240 min post milking and obtained 1,144 vocalizations (952 high frequency and 192 low frequency) (Gavojdian et al., 2024); the authors noted that calls were recorded under identical conditions and excluded noise. Another study analyzed 12 Holstein heifers and reported that vocalization rate peaked one interval before estrus climax and was higher during natural than induced estrus (Röttgen et al., 2018). (Yoshihara and Oya 2021) recorded 290 calls from 32 cows across four physiological states (feed anticipation, estrus, communication and parturition) and showed that call intensity, pitch and formant values reflected changes in salivary cortisol. (Katz 2020) captured 333 high frequency calls from 13 heifers and demonstrated that cows maintain individual vocal cues across contexts. Compared with these studies, our dataset contains both high and low frequency calls across positive and negative contexts, includes a richer set of behavioral classes (maternal, social, estrus, feeding, drinking, handling, distress, environmental and non vocal), and encompasses multiple farms and barn zones. This breadth enables analyses of behavioral diversity and cross context variation not previously possible.

Beyond scale and diversity, novelty also arises from the recording design, which is detailed in the next subsection.

### 2.2 Multi-microphone setup and multimodal synchronization

Recordings were collected from three commercial Holstein-Friesian dairy farms in Sussex County, New Brunswick, Canada over three consecutive days (5–7 May 2025), with one farm recorded per day between 9:00 am and 6:00 pm (Table 1). Across these sites, 65 raw audio files were obtained, representing a total of ~90 h of recordings (65.57 GB; mean file duration ~ 1 h 24 m, mean file size ~1.0 GB).

**Table 1:** Overview of recording sites and microphone-recorder setup.

**Farm ID**	**Herd size (Holstein/others)**	**Barn zones monitored**	**Microphones**	**Recorders**	**Raw files (n)**	**Total duration (h)**	**Total size (GB)**
Farm 1	57 Holstein	Feeding, drinking, milking, resting	Sennheiser MKH 416; RØDE NTG-2	Zoom F6; Zoom H4n Pro	21	~30 h	~21.5
Farm 2	207 Holstein/Jersey mix	Feeding, drinking, resting, milking	Sennheiser MKH 416; Zoom H4n Pro	Zoom F6; Zoom H4n Pro	21	~29 h	~22.0
Farm 3	160 Holstein	Feeding, resting, drinking	Wildlife bioacoustics recorder; RØDE NTG-2	Zoom F6; Zoom H4n Pro	23	~31 h	~22.1
**Total**	**424 cows**	All zones (feeding, drinking, milking, resting)	Multiple (MKH 416, NTG-2, Bioacoustics)	Zoom F6; H4n Pro;	**65**	**90 h 2 m**	**65.6**

Bold values indicate the total of all rows in the table.

**Figure 1:**
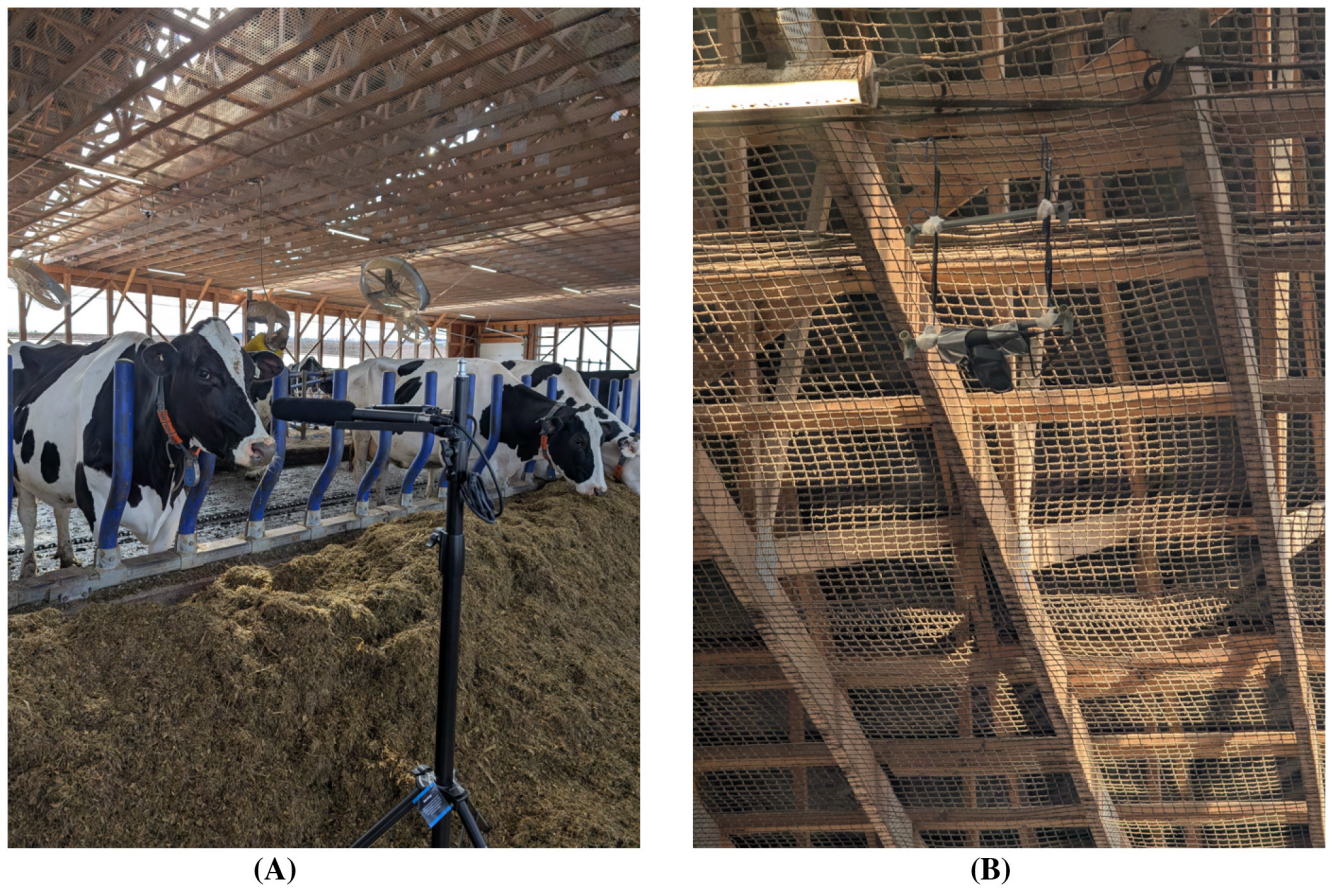
Multimicrophone and video setup used for synchronized multimodal recording in dairy barns. **(A)** Directional RØDE NTG-2 shotgun microphone connected to a Zoom H4n Pro portable recorder, deployed in the feeding section of farm. The setup was mounted on a stable tripod oriented toward the feed trough to capture high-fidelity vocalizations while rejecting off-axis barn noise. **(B)** Ceiling-mounted GoPro action camera positioned directly above the same zone in same farm to capture continuous 4K video of feeding behavior. The spatial alignment of microphone and camera ensured accurate cross-referencing between audio and behavioral context during manual annotation and ethological validation. **(A)** Feeding zone microphone setup. **(B)** Feeding zone camera setup.

The dataset captures four primary behavioral contexts—drinking, feeding, milking, and resting—within commercial barn environments. These settings included natural background sounds such as clanging metal gates, fans, tractors idling, overlapping vocalizations, and other routine noises. A representative example of the feeding-zone deployment is shown in Figure 1, where a shotgun microphone is co-located with a ceiling-mounted action camera to achieve synchronized audio-video capture of cow behavior at the feed trough. By integrating multiple farms and barn zones, the corpus reflects the true acoustic diversity of commercial dairy environments rather than controlled or laboratory conditions.

The novelty of this corpus lies in its multimicrophone, multi-sensor design. Unlike earlier bovine vocalization studies that relied on a single microphone or controlled settings, the present dataset integrates recordings from multiple farms, barn zones, and equipment types. Directional shotgun microphones (Sennheiser MKH 416, RØDE NTG-2) provided close-range, high signal-to-noise vocalizations, while portable recorders (Zoom H4n Pro, Zoom F6) and an autonomous Wildlife Bioacoustics logger captured longer-duration ambient soundscapes.

This design ensured that both focal vocal events (e.g., estrus calls, feeding anticipation) and background acoustic context (e.g., overlapping moos, barn machinery, human activity) were represented. The inclusion of video recordings from multiple GoPro cameras added a complementary visual dimension, enabling cross-referencing of acoustic events with behavioral context. Together, this multimodal, multi-sensor strategy produces a holistic, reproducible dataset that captures the acoustic reality of commercial dairy environments at unprecedented scale and resolution. This design also supports downstream statistical analyses that explicitly account for farm, barn zone, and microphone as potential sources of variance (Section 5.6), enabling users to quantify how much of the acoustic variability arises from environmental and equipment factors vs. behavioral class.

### 2.3 Environmental noise profiling

Dairy barns are acoustically challenging, with persistent machinery noise (milking robots, feeders, tractors), metal gate clanging, hoof impacts, people talking, urination, and wind. To enable robust vocalization extraction, separate noise recordings from each zone and analyzed using the Welch method with a 16,384-sample FFT window. The resulting noise inventory (Table 4) summarizes the spectral range and amplitude of different noise sources. Drinking noise exhibited low frequencies around 20 Hz and broad high-frequency peaks up to 1,029 Hz; the mean peak amplitude across samples was ~−40 dB. Feeding noise (mix of hisses, horns, and gate impacts) had low frequencies from 30 Hz and high-frequency components up to ~567 Hz with similar amplitude. Milking noise from robotic equipment was dominated by low frequencies near 12 Hz and high frequencies up to ~300 Hz and was louder (mean amplitude ~−22 dB). Resting noise (urination and human speech) spanned 12–493 Hz with mean amplitude ~−21 dB. These profiles informed the design of a band-pass filter (150–800 Hz) to remove low-frequency machinery noise and high-frequency electrical hiss while preserving the vocalization band. By providing a quantitative noise inventory, our dataset allows researchers to reproduce the preprocessing pipeline and evaluate the robustness of acoustic features against background noise.

### 2.4 Annotation scheme and ethological foundation

A key novelty of the dataset is its detailed annotation scheme (Table 2) informed by ethological principles and welfare protocols. The annotation system organizes vocalizations into nine main categories:

- Maternal & calf communication - includes calls such as Mother_Separation_Call (low frequency plaintive call when a cow is separated from her calf), Calf_Contact_Call (high frequency squeal when a calf seeks contact), and Maternal_Response_Call (mother cow responding back to her calves).
- Social recognition & interaction - encompasses affiliative calls like Greeting_Moo, Group_Contact_Call, Response_Exchange_Call, Herd_Coordination_Call and Social_Bonding, reflecting social hierarchy and cohesion.
- Estrus & mating behavior - includes Estrus_Call, characterized by loud high frequency bellowing signaling sexual receptivity; Mating_Excitement_Call; and Mounting_Associated_Call.
- Feeding & hunger related - covers Feed_Anticipation_Call (calls before feeding), Feeding_Fustration_Call, and Chewing_Rumination_Sounds (non vocal chewing noises).
- Water & thirst related - includes Drinking_Slurping_Sounds, Water_Anticipation_Call and Hydration_Distress.
- Distress & pain - covers High_Frequency_Distress (intense distress vocalizations), Frustration_Call, Injury_Pain_Moo, Sneeze, Cough and Burp.
- Environmental & situational - includes vocal responses to environmental stimuli like Weather_Response_Call, Transportation_Stress_Call and Confinement_Protest_Call.
- Non vocal sounds - comprises non vocal behaviors such as Breathing_Respiratory_Sounds and Licking_Sounds.

**Table 2:** Mapping of acoustic features to biological interpretation and representative call types in the dataset.

**Acoustic feature**	**Biological interpretation**	**Example call types**
Fundamental frequency (F0: mean, min, max)	Determined by vocal fold tension, length, and mass; high F0 reflects arousal, distress, or estrus, while low F0 indicates calm affiliative contact.	*Estrus_Call, High_Frequency_Distress*, low-frequency moos (contact)
Formant frequencies (F1, F2)	Resonances of the vocal tract linked to mouth opening, tongue/lip position, and body-size cues; larger F1–F2 separation often accompanies noisier or less harmonic structure.	*Water_Slurping_Sounds*, harmonic moo (stable formants)
Duration & timing (Start, End, Duration)	Persistence of calling; shorter calls often reflect neutral/positive states, whereas longer calls are associated with higher arousal or separation.	*Mother_Separation_Call* (long, low intensity); *Feed_Anticipation_Call* (short bursts)
Energy measures (RMS, intensity, time to peak)	Reflect call forcefulness and emotional valence; high RMS and fast time-to-peak indicate urgency, while low intensity with long duration suggests persistent, subdued calls.	*Aggressive_Bellow, Frustration_Call*; calf contact moo (low intensity)
Spectral centroid, bandwidth, roll-off, Zero-Crossing Rate (ZCR)	Differentiate harmonic vs. noisy events; high centroid/ZCR imply noisy or aperiodic content, low centroid indicates harmonic structure.	Sneezes, burps (high centroid, high ZCR); harmonic moo (low centroid, low ZCR)
Mel-Frequency Cepstral Coefficients (MFCCs)	Capture global spectral shape and timbre; useful for subtle distinctions between similar call types, with MFCC-based estrus detection reported at >90% accuracy.	*Feed_Anticipation_Call* vs. *Feeding_Frustration_Call*
Voiced ratio	Proportion of voiced vs. unvoiced frames; distress calls tend to include more unvoiced segments, whereas nasal moos are almost fully voiced.	High-frequency distress calls (more unvoiced); nasal moos (fully voiced)

Each sub category is accompanied by a concise description (e.g., Estrus_Call is a prolonged high frequency call emitted by a receptive female; Feed_Anticipation_Call is a rhythmic moo produced when cows expect feeding). The categories deliberately span positive and negative welfare states in line with welfare science. Prior research supports this ethological structuring: high frequency calls with the mouth open are associated with distress or long distance communication, whereas low frequency calls with the mouth closed occur in calm social contexts (Jobarteh et al., 2024). Vocalization rates also provide behavioral cues—for example, (Röttgen et al. 2018) found that call rate peaks before estrus climax, and (Yoshihara and Oya 2021) linked increased formant frequencies to elevated cortisol during parturition. By capturing a wide range of vocal types and non vocal sounds, our annotation scheme enables analysis of both emotional valence and arousal, as recommended in contemporary animal welfare frameworks.

### 2.5 Rich metadata and FAIR compliance

The dataset is accompanied by a comprehensive metadata table (Table 7) describing each clip. Key fields include the unique file name, recording date, farm identifier, barn zone, microphone model, duration, pitch statistics (mean, minimum and maximum), and formant frequencies (F1 and F2), along with categorical annotations such as main category, subcategory, emotional context, confidence score, and textual description. Feature definitions follow acoustic and ethological conventions–for example, pitch relates to laryngeal tension and arousal, formant spacing reflects vocal tract length, and energy measures indicate call strength.

The metadata structure aligns with the FAANG (Functional Annotation of Animal Genomes) guidelines for animal metadata and adheres to the FAIR principles (Harrison et al., 2018). These principles ensure that datasets are described with rich metadata, use community standards, and are stored in formats that facilitate long-term reuse across research communities. Applying FAIR to bioacoustic corpora enhances transparency, reproducibility, and integration with other animal genomics and welfare datasets (Harrison et al., 2018).

Overall, the dataset's novelty lies in its scale, diversity, multi-microphone design, multimodal referencing, detailed ethology-driven annotation scheme, and metadata structure that complies with international standards. Compared with previous bovine vocalization studies that focused on small cohorts or narrow behavioral contexts, this corpus offers a more holistic and reproducible resource for advancing machine learning and welfare research in dairy cattle. Having established the dataset's scope and comparative novelty, we now describe the data collection process, including recording sites, equipment, and behavioral context capture.

## 3 Data collection

### 3.1 Recording sites

Data were collected from three commercial dairy farms in Sussex County, New Brunswick, coded FARM1-FARM3, all of which operated free-stall barns (Table 1). Farm 1 housed 57 Holstein cows, Farm 2 housed 207 cows in a mixed Holstein-Jersey herd, and Farm 3 housed 160 Holstein cows. Recording took place during sequential site visits (5–7 May 2025), ensuring coverage across farms under comparable seasonal conditions.

Each barn was divided into four monitored zones—drinking troughs, feeding alleys, milking parlor, and resting pens. Within each zone, microphones were installed at fixed mounting locations (for example above or beside water bowls, near feed mangers, or along the fronts of resting stalls) chosen to coincide with areas where cows repeatedly congregate. Rather than enforcing a single, fixed source-microphone distance, this design reflected how animals naturally move through these spaces. As cows approached, fed, drank, queued for milking, or lay down, their vocalizations were typically produced within a practical distance range of approximately 0.5–3 m from the nearest microphone, depending on their momentary position and orientation. This zone-based layout enabled simultaneous, zone-specific recording and provided systematic contrasts between different acoustic environments such as feeding areas, milking stations, and resting pens.

In addition, manual observation logs were maintained throughout, noting events such as feeding schedules, veterinary visits, or machinery maintenance. These logs ensured that contextual events were linked to the acoustic data, creating a diverse soundscape that is representative of everyday husbandry practices in Atlantic Canadian dairy barns.

To capture these environments effectively, a multimicrophone hardware setup was deployed, as described below.

### 3.2 Recording hardware and microphone placement

To ensure representative acoustic coverage, a multimicrophone array was deployed, combining directional shotgun microphones with portable recorders and one autonomous bioacoustics logger:

- Sennheiser MKH 416—hypercardioid interference-tube shotgun microphone, widely used in film and wildlife recording. Its strong side rejection allowed capture of subtle vocal nuances despite barn noise (Sennheiser, 2023).
  – Paired with Zoom F6—six-channel portable recorder powered via phantom supply. The F6 supported 32-bit float recording, dual A/D converters, and ultra-low-noise preamps, preventing clipping even during high-intensity calls (Zoom, 2023).
- RØDE NTG-2—supercardioid shotgun microphone, battery/phantom powered, valued for affordability and portability, suited for close-range recordings in drinking and milking contexts (RØDE, 2023).
  – Paired with Zoom H4n Pro—four-track handheld recorder powered by two internal AA rechargeable batteries. The H4n Pro included built-in X/Y stereo microphones, dual XLR inputs, and 24-bit recording with maximum SPL handling of 140 dB (Zoom, 2022). Two such RØDE NTG-2 + H4n Pro pairs were deployed for zone-specific coverage.
- Wildlife Bioacoustics autonomous recorder—a single passive logger programmed for scheduled monitoring, especially in resting areas. It operated continuously on four AAA rechargeable batteries, enabling long-duration capture without human presence (Wildlife Acoustics, 2023).

**File characteristics:** Zoom F6 recordings were largest on average (~1.7 GB, mean peak amplitude −43.7 dB, 20–1,029 Hz range), followed by Zoom H4n Pro (~1.6 GB, −30.5 dB, 20–820 Hz). The autonomous logger produced smaller files (~308 MB, −20.1 dB, 12–893 Hz). By context, the drinking zone generated the largest raw data volume (~3.65 GB), followed by feeding (~1.7 GB), milking (~762 MB), and resting (~450 MB).

**Cameras:** Five fixed GoPro action cameras were installed above barn zones, recording continuously at 4K/30 fps with wide-angle lenses to cover feeding alleys, resting pens, drinking troughs, and the milking parlor. Cameras were synchronized with audio via a shared timecode feed.

**Figure 2:**
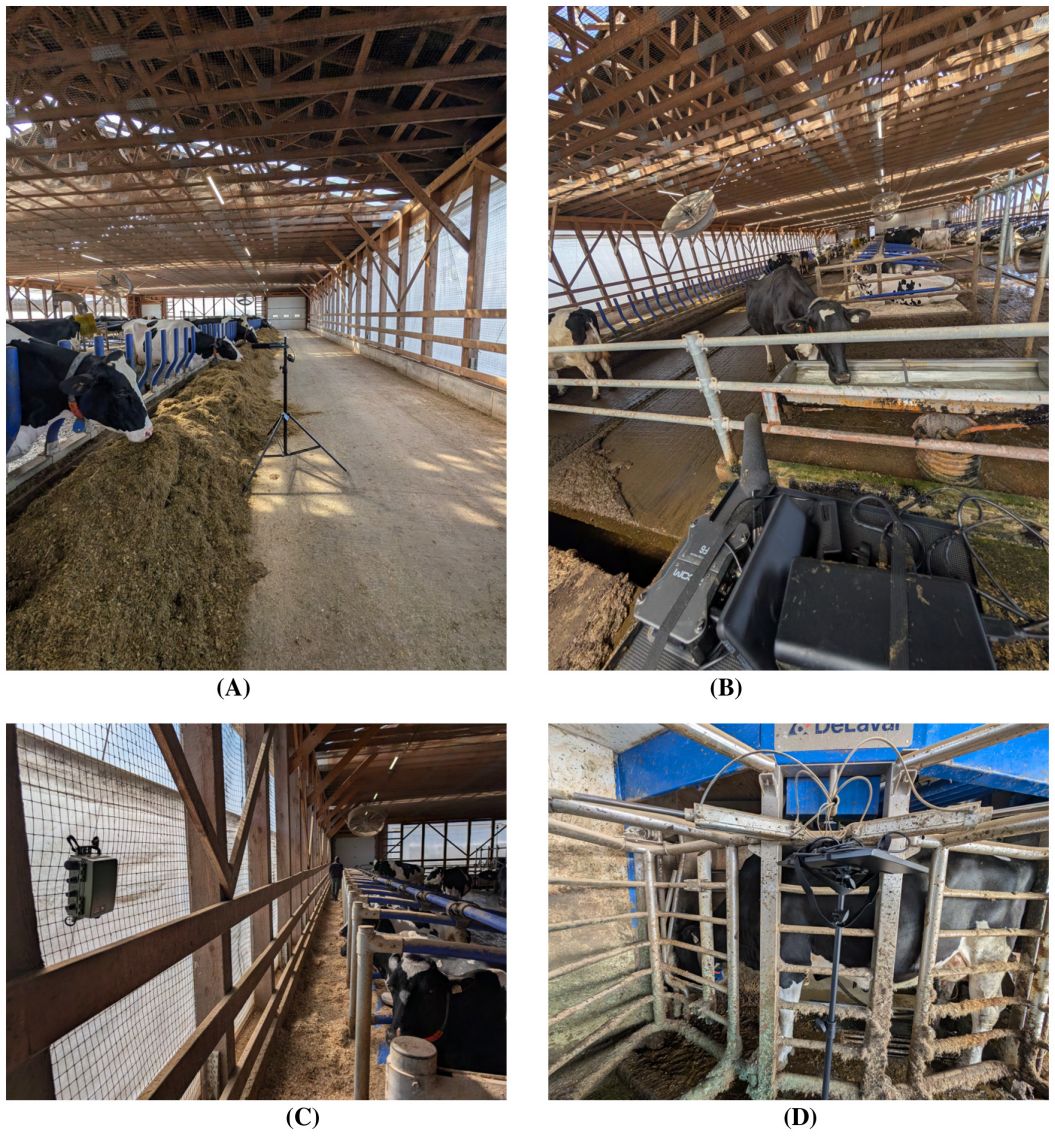
Microphone setups across barn zones at Farm 1. **(A)** RØDE NTG-2 shotgun microphone positioned along the feeding alley to capture close-range vocalizations during feeding activity while minimizing side reflections. **(B)** Sennheiser MKH 416 directional microphone placed near the drinking trough to record high-clarity vocal and non-vocal events amid tractors and metallic noise. **(C)** Autonomous Wildlife Bioacoustics recorder installed in the resting zone to capture low-frequency moos and background group vocalizations without human presence. **(D)** RØDE NTG-2 with Zoom H4n Pro handheld recorder positioned near the milking station to document vocal and non-vocal sounds associated with handling and milking routines.

**Placement strategy:** Microphones were mounted on adjustable stands ~ 1 m above cow head height, oriented toward the zone center. This prevented contact with animals, minimized wall reflections, and ensured zone-specific capture. Cables were routed along beams and tripods stands and shielded to prevent chewing. The multimicrophone array (Figure 2) enabled concurrent multi-zone recording and cross-comparison of calls across environments.

This combination of hardware provided complementary perspectives: phantom-powered Sennheiser + Zoom F6 setups for high-fidelity focal recording, AA-powered RØDE + Zoom H4n Pro pairs for flexible mobile coverage, and the AAA-powered Wildlife Acoustics logger for unattended long-term monitoring. Together with parallel video capture, the setup preserved both individual-level vocal features and group-level acoustic context, forming a robust foundation for behavioral and machine-learning analyses.

The full microphone and recorder specifications, including deployment zones, recording durations, file sizes, and frequency ranges for each configuration, are summarized in Table 3. Because cows moved freely within these monitored zones, the effective source-microphone distance varied naturally from call to call. In practice, vocalizations were usually produced when cows were at, or passing through, the focal locations (feed rail, water bowl, parlor entry, stall front), leading to a typical distance range of around 0.5-3 m from the microphone. This variability was intentional: the aim was to reproduce the acoustic conditions under which real farm monitoring systems would operate, rather than forcing a laboratory-style fixed geometry.

**Table 3:** Microphone and recorder setup used for barn vocalization recordings.

**Microphone / Recorder**	**Key specifications**	**Deployment zone(s)**	**Rationale in barn context**	**Avg. size (MB)**	**Avg. duration**	**Peak (dB)**	**Freq. range (Hz)**
Sennheiser MKH 416 (shotgun) + Zoom F6	Hypercardioid, interference tube; 40–20,000 Hz; paired with 32-bit float recorder	Feeding alleys, resting areas	Extremely directional; avoids barn noise and prevents clipping during loud moos	~1,738	~1 h 28 m	–43.7	20–1,029
RØDE NTG-2 (shotgun) + Zoom H4n Pro	Supercardioid; 20–20,000 Hz; paired with 24-bit recorder	Drinking troughs, milking parlor	Affordable, portable; good for close-up calls with reduced side interference	~1,555	~1 h 25 m	–30.5	20–820
Zoom H4n Pro (built-in XY + XLR inputs)	Handheld, stereo + external inputs; 24-bit/96 kHz	Feeding and milking	Mobile capture, flexible for focal recording	–	–	–	–
Zoom F6 (standalone channels)	6-channel, 32-bit float, dual A/D converters	Feeding/resting	Long sessions with wide dynamic range	–	–	–	–
Wildlife Acoustics logger	Passive autonomous system; duty-cycled	Resting pens, background	Continuous scheduled monitoring without human presence	~308	~1 h 15 m	–20.1	12–893

The directional characteristics of the MKH 416 and NTG-2 partially compensate for distance-related attenuation by preferentially capturing sounds within their frontal pickup patterns and suppressing much of the off-axis machinery and barn noise. At the analysis stage, our feature extraction pipeline includes amplitude normalization and z-score standardization, which reduces the influence of absolute intensity differences arising from distance variation. Moreover, the majority of the 24 acoustic features used in subsequent models–such as fundamental frequency, formant ratios, spectral centroid, and MFCCs–describe the shape and structure of the signal rather than its absolute amplitude, and are therefore relatively robust to moderate changes in distance.

Together, the zone-based, fixed microphone positions and distance-robust feature design ensure that the dataset captures realistic variability in recording conditions while remaining suitable for machine-learning applications.

### 3.3 Behavioral context capture

Audio alone rarely conveys the full meaning of vocalizations. To provide behavioral context, the project implemented a multimodal capture protocol. Behavioral video was recorded using the fixed GoPro cameras described above, and three researchers maintained manual notes documenting the time of day, weather conditions, feeding schedules, milking events, and notable social interactions or stressors between cows. The annotation framework was informed by Tinbergen's four questions (Bateson and Laland, 2013), which remain central in animal behavior research. In the context of cow vocalization, these can be adapted as follows:

- **Function** (adaptive value): What role does a call serve in daily life? For example, does it facilitate feeding coordination, signal distress, or attract social attention?
- **Phylogeny** (evolutionary background): How do vocal traits in dairy cattle relate to those seen in other bovids or domesticated species?
- **Mechanism** (causation): What immediate physiological or environmental factors trigger a vocalization (e.g., hunger, pain, separation, handling, or barn noise)?
- **Ontogeny** (development): How does vocal behavior vary with age, parity, or experience (e.g., heifers vs multiparous cows)?

By linking proximate mechanisms (physiology, environment) with ultimate functions (communication, adaptation), this framework strengthens interpretation of the dataset beyond acoustics alone. For instance, high-frequency open-mouth calls may be tied to immediate arousal or stress, while also serving long-term communicative roles within the herd (Jobarteh et al., 2024).

Audio-video alignment was performed manually. Instead of using automated synchronization hardware, researchers cross-referenced the timestamps of audio recordings with video footage and their own observation logs. This practical approach enabled vocal events to be matched with visible behaviors (such as feeding, resting, or responding to handling) without specialized tools.

### 3.4 File handling and storage

Field data were initially stored on the internal memory cards of the Zoom recorders and GoPro cameras. To prevent overwriting or accidental data loss, recordings from each farm were transferred immediately after the day's data collection. Files were copied to a secure laptop on-site and then uploaded to a shared Dropbox repository, ensuring both immediate backup and remote accessibility. Audio recordings were saved in WAV format (44.1 or 48 kHz, 24-bit depth) and named according to a structured convention: FarmID - MicrophonePlacement - BarnZone - Date - Time. Video files were stored in MP4 format with matching time stamps to maintain cross-referencing with audio.

To safeguard data integrity, the raw original files remain archived in Dropbox. For subsequent analysis steps such as preprocessing, cleaning, and segmentation, working copies were downloaded and processed locally, ensuring that the original dataset was preserved without modification. Metadata spreadsheets were updated during each transfer to log file names, times, equipment used, and backup status. This multi-stage handling approach–memory card → laptop → cloud backup → working copies–provided a robust and traceable workflow that minimized the risk of data loss and maintained strict separation between raw and processed datasets.

### 3.5 Data processing workflow

**Figure 3:**
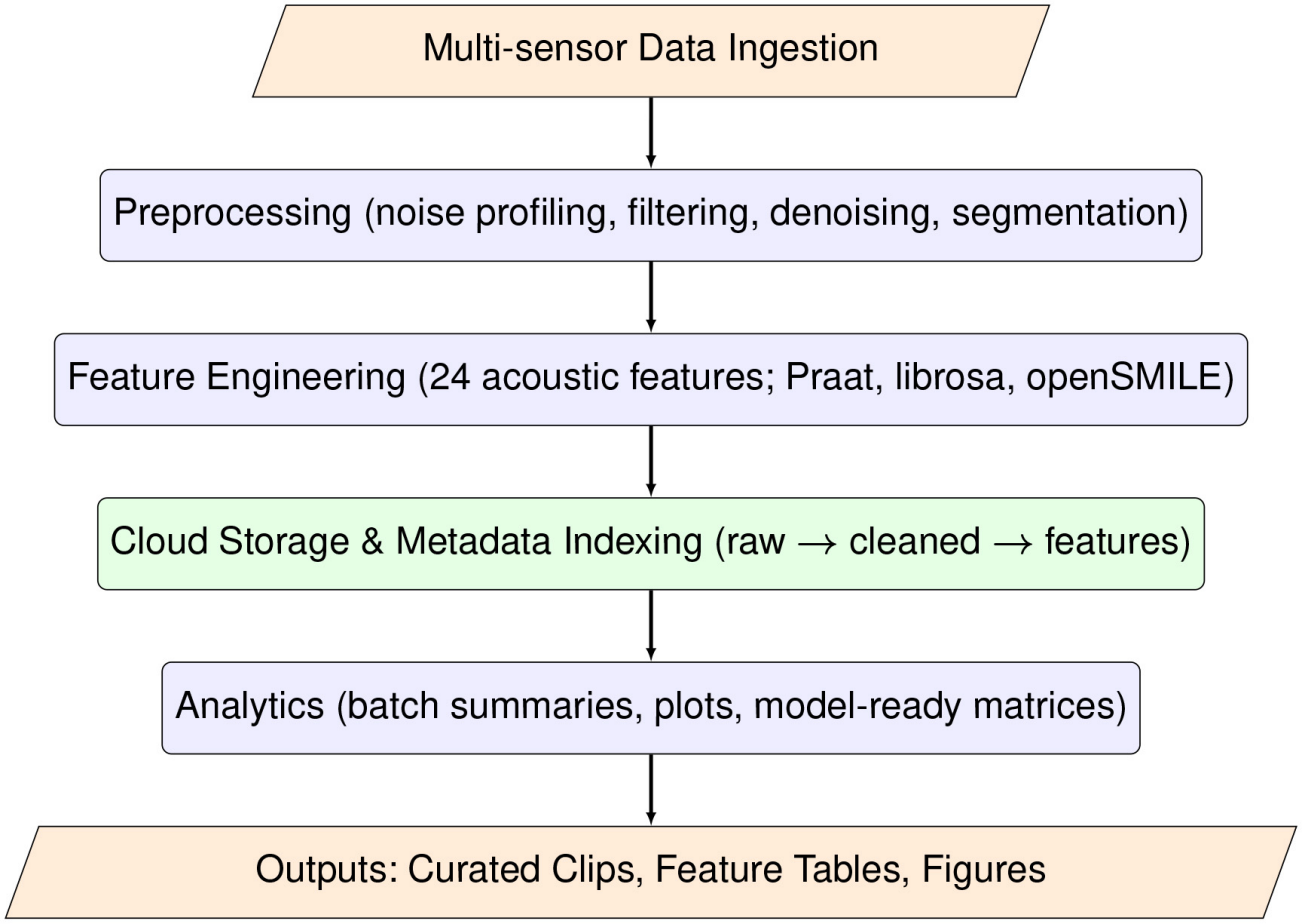
Modular data processing workflow for the bovine bioacoustics dataset, showing the sequential stages of data ingestion, preprocessing (filtering and denoising), manual segmentation, acoustic feature engineering, storage and metadata indexing, and downstream analytics and machine-learning.

To efficiently manage and analyse the ~ 90 h of multimodal recordings (65 raw files; 65.6 GB), a modular and reproducible data processing workflow was established in alignment with FAIR and FAANG principles. Rather than a fully distributed computing system, this workflow is best understood as a set of clearly defined batch-processing stages that can be scaled to distributed or cloud-based frameworks in future work. The workflow (Figure 3) integrates five sequential stages, from ingestion to analytics, ensuring traceable and scalable processing of livestock bioacoustic data.

Processing was performed on a workstation equipped with an Intel Core i9-12900K processor (16 cores, 24 threads), 64 GB RAM, and an NVIDIA RTX 3090 GPU (24 GB VRAM). Primary storage consisted of a 4 TB NVMe SSD, with continuous cloud backup to a 2 TB Dropbox repository for redundancy and remote access. All core steps were implemented in Python 3.10 using open-source libraries, ensuring that the workflow can be replicated without access to proprietary software. The workflow comprises the following components:

- **Data ingestion**—Multi-sensor audio and video streams from the three farms were organized using a structured, time-stamped file-naming convention encoding farm, barn zone, date, time, and device ID. Raw WAV files (44.1/48 kHz, 24-bit) were ingested via Python scripts that used librosa for audio input/output and pandas for logging file-level metadata. This automated ingestion step standardized file formats, checked for corrupted or incomplete recordings, and populated metadata tables for downstream processing.
- **Preprocessing**—Raw recordings were processed in batches following the standardized steps outlined in Sections 4.1-4.4, including spectral noise profiling, band-pass filtering, adaptive denoising, manual segmentation, and acoustic verification. Batch preprocessing was implemented in Python 3.10 to standardize signal quality across the heterogeneous barn environments. We applied a fourth-order Butterworth band-pass filter (50–1,800 Hz) using scipy.signal to isolate the frequency range relevant for bovine vocalizations and attenuate low-frequency machinery noise and high-frequency artifacts. Spectral gating-based denoising was then performed with the noisereduce library to suppress non-stationary background noise while preserving vocal structure. In parallel, manual segmentation was carried out in Raven Lite 2.0, using the spectrogram and synchronized video to identify individual vocalization events and exclude purely mechanical or non-vocal sounds.
- **Feature engineering**—for each segmented clip, we derived a 24-dimensional acoustic feature vector designed to capture the key temporal, voicing, spectral, and cepstral properties of cattle vocalizations. Core source-related features (fundamental frequency, formant frequencies, intensity, and harmonic-to-noise ratio) were extracted using Parselmouth, a Python interface to Praat. Complementary spectral descriptors such as spectral centroid, bandwidth, roll-off, root-mean-square (RMS) energy, and zero-crossing rate, along with Mel-frequency cepstral coefficients (MFCCs), were computed using librosa (version 0.10.1). For cross-validation and extended feature sets, we additionally used openSMILE (version 2.3.0).
- **Storage and metadata indexing**—Cleaned audio clips, feature tables, and contextual metadata were stored in a structured directory hierarchy that clearly separated raw and processed data and supported long-term reuse. Original recordings were archived in a /raw folder, denoised and filtered clips were stored under /processed, per-clip feature tables were saved in /features, contextual and schema information in /metadata, and annotation files (e.g., labels, time boundaries) in /annotations. This layout ensured that every processed object could be traced back to its raw source file and associated metadata.
- **Analytics and output generation**—Aggregated feature tables were analyzed using pandas and scipy.stats to perform descriptive statistics and inferential tests, including Kruskal-Wallis tests and Dunn's post-hoc comparisons. Visualizations such as violin plots, spectrogram panels, and Pareto charts were generated with matplotlib and seaborn to characterize class-wise distributions and dataset structure. Machine-learning models were trained and evaluated using scikit-learn (version 1.3.0) and PyTorch (version 2.0), enabling both classical and deep-learning approaches to be applied to the same standardized feature sets.

#### 3.5.1 Scalability considerations

Although all analyses in this study were conducted on a single workstation, the modular design of the workflow facilitates future scaling. Ingestion, preprocessing, and feature extraction stages can be parallelised using frameworks such as Apache Spark or Dask; storage can be migrated to cloud object stores such as Amazon S3 or Google Cloud Storage for multi-site deployments; and the entire pipeline can be containerised using Docker to support reproducible execution across different computing environments.

With raw recordings secured across farms and barn zones, the following Section 4 details the preprocessing pipeline applied to enhance signal quality and prepare clips for segmentation and annotation.

### 3.6 Ethical approvals

All experimental procedures were reviewed and approved by the Dalhousie University Animal Ethics Committee (Protocol No. 2024–026). Data collection involved no physical interaction with animals, and all participating farm owners were fully informed of the study's objectives and provided written consent. In accordance with institutional and national ethical standards, data were obtained solely through passive audio, image, and video recordings.

## 4 Data preprocessing

### 4.1 Noise profiling

Noise spectral profiling was carried out as the first stage of preprocessing, since raw barn recordings contained a wide range of background sounds from machinery, metal gates, hoof impacts, people, and other animals. This process involved analyzing background noise patterns in terms of frequency (Hz) and amplitude (dB) in order to distinguish cow vocalizations from environmental sources. Noise-only segments were extracted from the recordings for each barn zone and analyzed using Audacity with the Welch spectrum function, configured with a 16,384-point FFT window, 50% overlap, and a logarithmic frequency axis. The Welch method was chosen because it averages overlapping segments, giving smoother spectra and suppressing transient spikes. Alternative methods such as Bartlett (less smoothing), Blackman-Harris (suited for controlled studio audio), and Hanning (unstable under noisy barn conditions) were considered, but Welch proved to be the most reliable for real-world farm recordings. The analysis revealed distinct noise signatures for different barn zones.

- Drinking areas were dominated by metallic clanging of bowls, splashing at troughs, and bowel noise, with frequency peaks extending up to around 1029 Hz and mean amplitudes of approximately -60 dB.
- Feeding zones produced a mixture of metallic impacts, compressed air hisses, and horn-like sounds, spanning 30–300 Hz with variable amplitudes.
- Milking parlors were characterized by robotic systems, pumps, and vacuum lines, which generated relatively narrow frequency bands around 100–200 Hz but at higher amplitudes between -10 and -36 dB.
- Resting areas contained low-frequency components between 25–80 Hz from urination, rumination, and equipment hum, combined with higher-frequency sounds such as people talking.
- In addition, microphone hiss was consistently detected below 50 Hz across all farms and zones.

These findings were consistent with the expectation that barn-specific activities and equipment each contribute distinctive background noise signatures that overlap with the vocal frequency space of cows.

**Figure 4:**
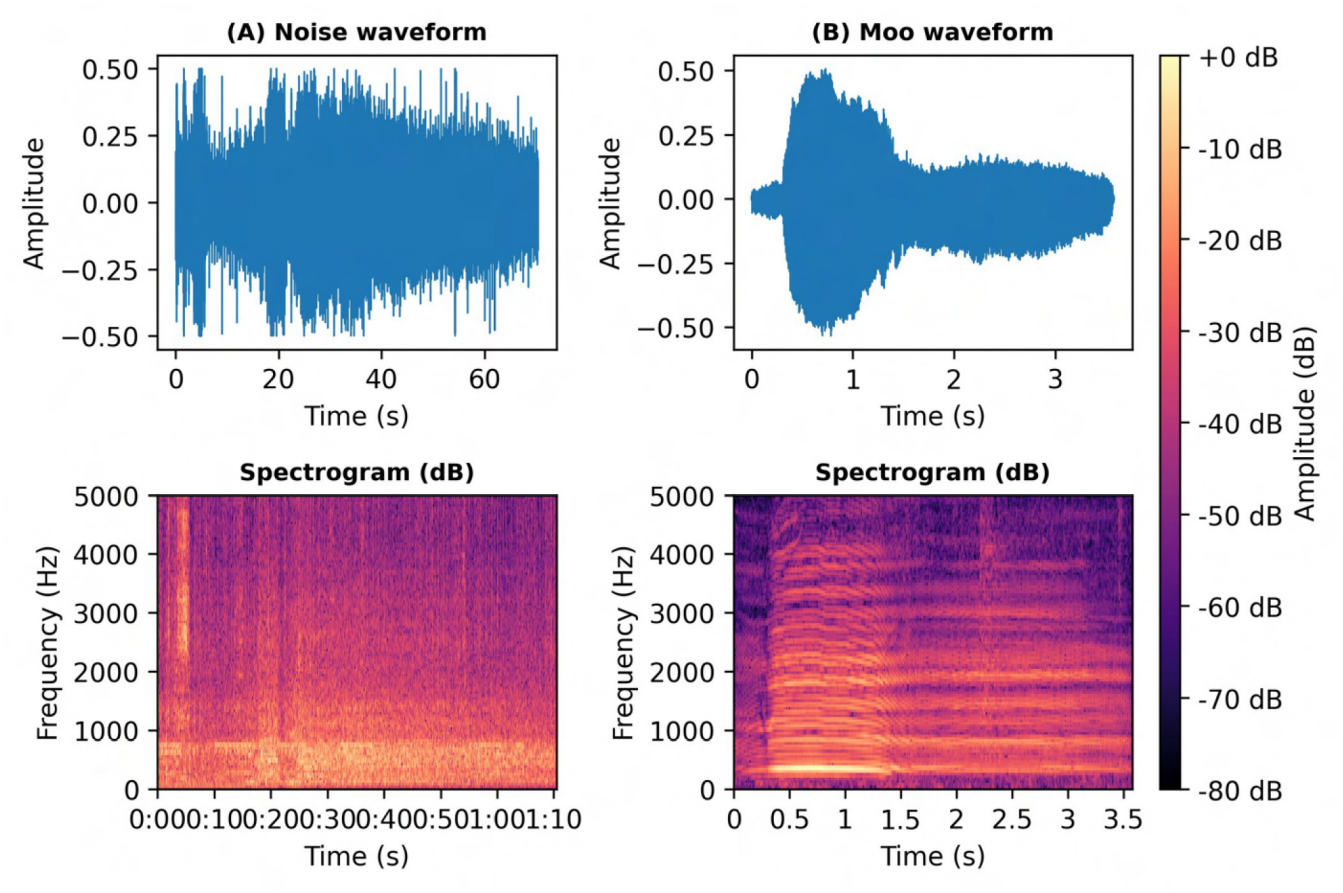
Comparison of barn noise and cow vocalization spectrograms. **(A)** Waveform and spectrogram of a barn noise segment dominated by broadband mechanical and environmental energy. **(B)** Waveform and spectrogram of a cow “moo” call recorded in the same acoustic environment, showing harmonic stacks and formant bands with periodic energy peaks. Spectrograms were computed using short-time Fourier transform and displayed in decibel scale relative to the maximum amplitude to enable direct visual comparison of spectral structure and signal clarity.

The profiles are summarized in Table 4, which presents the farm-wise and barn-zone-wise distribution of noise sources, frequency ranges, amplitudes, and the microphones used. This information directly informed the design of the filtering pipeline applied in subsequent preprocessing, where band-pass filtering was configured to retain the main vocal range (~ 100–1,800 Hz) while attenuating machinery hum, human speech, and high-frequency hiss. A representative spectrogram and waveform comparison (Figure 4) contrasts a cow vocalization with a noise-only segment, illustrating the necessity of noise profiling before segmentation and feature extraction. These zone-specific spectral noise profiles then directly informed the parameter choices for band-pass filtering and spectral denoising in the subsequent preprocessing stages (Sections 4.2–4.4).

**Table 4:** Farm-wise noise spectral profiles across barn zones.

**Day**	**Farm**	**Category**	**Noise source**	**Low frequency**	**High frequency**	**Peak amplitude**	**Microphone**
Day 1	Farm 1	Drinking	Metallic plates	190 Hz	655 Hz	−60.5 dB	Zoom F6
Day 1	Farm 1	Feeding	Bowel movement	229 Hz	567 Hz	−54.8 dB	Zoom H4n
Day 1	Farm 1	Milking	Robotic milking system	106 Hz	127 Hz	−32.2 dB	Zoom H4n
Day 1	Farm 1	Resting	Tractor	103 Hz	120 Hz	−1.8 dB	Bioacoustics
Day 2	Farm 2	Drinking	Birds chirping	421 Hz	579 Hz	−25.7 dB	Zoom H4n
Day 2	Farm 2	Feeding	Metal plates	164 Hz	283 Hz	−58.2 dB	Zoom F6
Day 2	Farm 2	Milking	Robotic milking system	217 Hz	301 Hz	−25.7 dB	Zoom H4n
Day 2	Farm 2	Resting	Microphone hiss	12 Hz	19 Hz	−13.9 dB	Bioacoustics
Day 3	Farm 3	Drinking	Hiss + urination sound	91 Hz	146 Hz	−24.6 dB	Zoom H4n
Day 3	Farm 3	Feeding	Hiss + tractor horn	30 Hz	72 Hz	−14.2 dB	Zoom F6
Day 3	Farm 3	Milking	People talk + milking system	100 Hz	130 Hz	−5.4 dB	Bioacoustics
Day 3	Farm 3	Resting	Hiss	25 Hz	38 Hz	−20.3 dB	Zoom H4n

### 4.2 Band pass filtering

Based on the results of the noise spectral profiling and the known frequency ranges of bovine vocalizations, a fourth-order Butterworth band-pass filter was applied with cut-off frequencies set at 50 Hz and 1,800 Hz. This frequency range was selected to retain the majority of bovine vocal energy–where the fundamental frequency typically lies between 100 and 300 Hz and harmonics extend up to approximately 1,000 Hz–while attenuating low-frequency machinery hum below 50 Hz and high-frequency electrical hiss above 1,800 Hz. Previous studies have reported comparable ranges, noting that cattle vocalizations commonly exhibit fundamental frequencies between 80 and 180 Hz for cows and calves, with energy extending up to 1 kHz or higher in certain contexts (de la Torre et al., 2015; Briefer, 2012; Lenner et al., 2025). The Butterworth filter was chosen due to its maximally flat frequency response in the passband, which avoids distortion of harmonic structure and preserves acoustic fidelity. Its use is well established in acoustic and bioacoustic research as a robust method for isolating biologically relevant frequency bands (MacCallum et al., 2011; Simmons et al., 2022). To eliminate phase shifts that might affect later acoustic feature extraction, such as pitch contour or spectral energy analysis, filtering was implemented using zero-phase forward-backward filtering with the butter and filtfilt functions from Python's scipy.signal library.

This filtering step plays a critical role in the preprocessing pipeline. By suppressing spectral energy outside the vocalization band, it reduces broadband interference and makes the harmonic patterns of vocalizations stand out more clearly in spectrograms. This is particularly important in barn environments where background noise from ventilation systems, metallic clanging, and robotic milking machinery often overlaps with vocal frequencies in the 50–1,800 Hz range. While some overlap remains, the band-pass filtering substantially improves the signal-to-noise ratio, emphasizing the stable formants of cow vocalizations, which are most prominent between 200 and 400 Hz (Watts and Stookey, 2000; Green et al., 2019).

The filtering process also generates two outputs. First, spectrogram data are exported as a CSV file, providing frequency bins over time in tabular form, which enables automated detection of vocal events such as moos, sneezes, or coughs without continuous manual listening. Second, filtered WAV audio files are produced, which present reduced background hiss and rumble and therefore facilitate cleaner listening and manual annotation. Together, these outputs provide both a human-audible and machine-readable foundation for subsequent stages of analysis.

**Figure 5:**
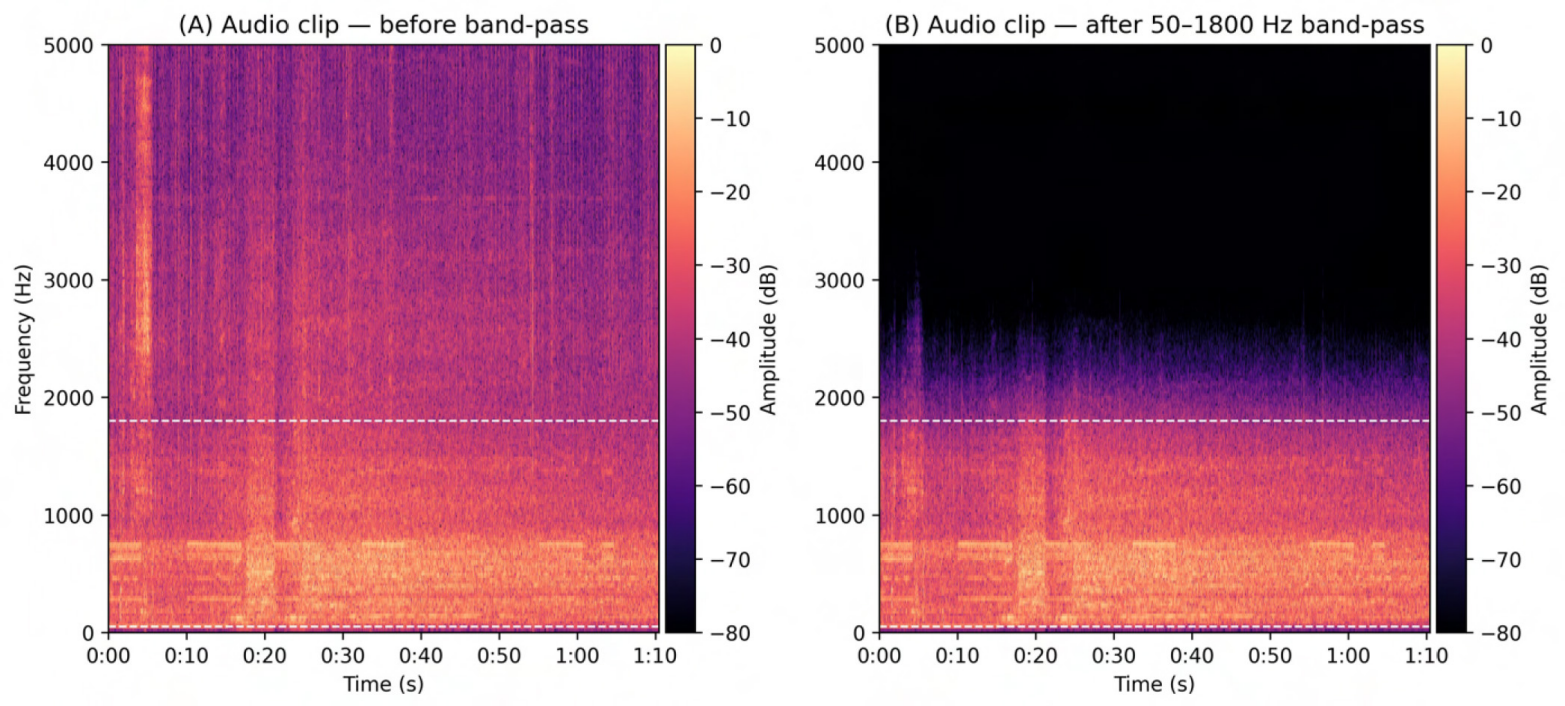
Effect of band-pass filtering on an audio clip. **(A)** Spectrogram of the unfiltered signal showing broadband energy, including low-frequency hum and high-frequency hiss. **(B)** Spectrogram after 50–1,800 Hz band-pass filtering, with clear attenuation of energy below 50 Hz and above 1.8 kHz while preserving in-band harmonic structure. Dashed lines mark the passband; both panels share identical time and frequency axes (displayed to 5 kHz) and a common dB color scale for direct visual comparison.

Although this step does not entirely eliminate overlapping barn noise within the vocalization band, it substantially improves clarity and prepares the audio for further visualization, labeling, and feature extraction. A side-by-side comparison of pre- and post-filtering spectrograms (Figure 5) illustrates the suppression of broadband noise while preserving the harmonic structure of cow calls.

### 4.3 Noise reduction in iZotope RX 11

Following band-pass filtering, additional noise suppression was carried out using iZotope RX 11 (iZotope Inc., Cambridge, MA), a professional-grade audio restoration suite that offers fine-grained spectral editing, adaptive noise learning, and fault repair modules. Although iZotope RX is not yet cited in published bioacoustic studies, its functionality parallels classical spectral denoising and repair techniques long used in animal sound research (e.g. spectral subtraction, MMSE spectral estimators, wavelet denoising) (Xie et al., 2021; Brown et al., 2017; Juodakis and Marsland, 2022). More recently, methods such as Biodenoising adopt high-quality pre-denoising (often via speech-based or spectral tools) as pseudo-clean references for training animal-specific models, which further validates the use of advanced denoising tools upstream. RX 11 was chosen because it allows the user to visually inspect the time-frequency structure, select noise-only regions, build adaptive noise profiles, and non-destructively subtract noise while preserving the vocal harmonics of interest. This flexibility is especially useful in barn settings, where noise is heterogeneous (e.g. ventilation hum, mechanical clanks, electrical hiss) and overlaps in frequency with vocal energy.

We implemented a multi-stage noise reduction pipeline consisting of the following steps:

1. Gain normalization and DC offset removal to standardize amplitude baselines across recordings.
2. Spectral De-noise to compute adaptive noise estimates from silent (non-vocal) segments and subtract them from the signal.
3. Spectral Repair to mitigate transient artifacts (e.g., gate slams or rapid mechanical clicks) by interpolating across missing time-frequency bins.
4. De-clip and De-crackle modules to correct occasional saturation and impulsive noise events.
5. EQ Match to smooth the overall frequency response and compensate for microphone coloration, resulting in a more natural tonal balance.

**Figure 6:**
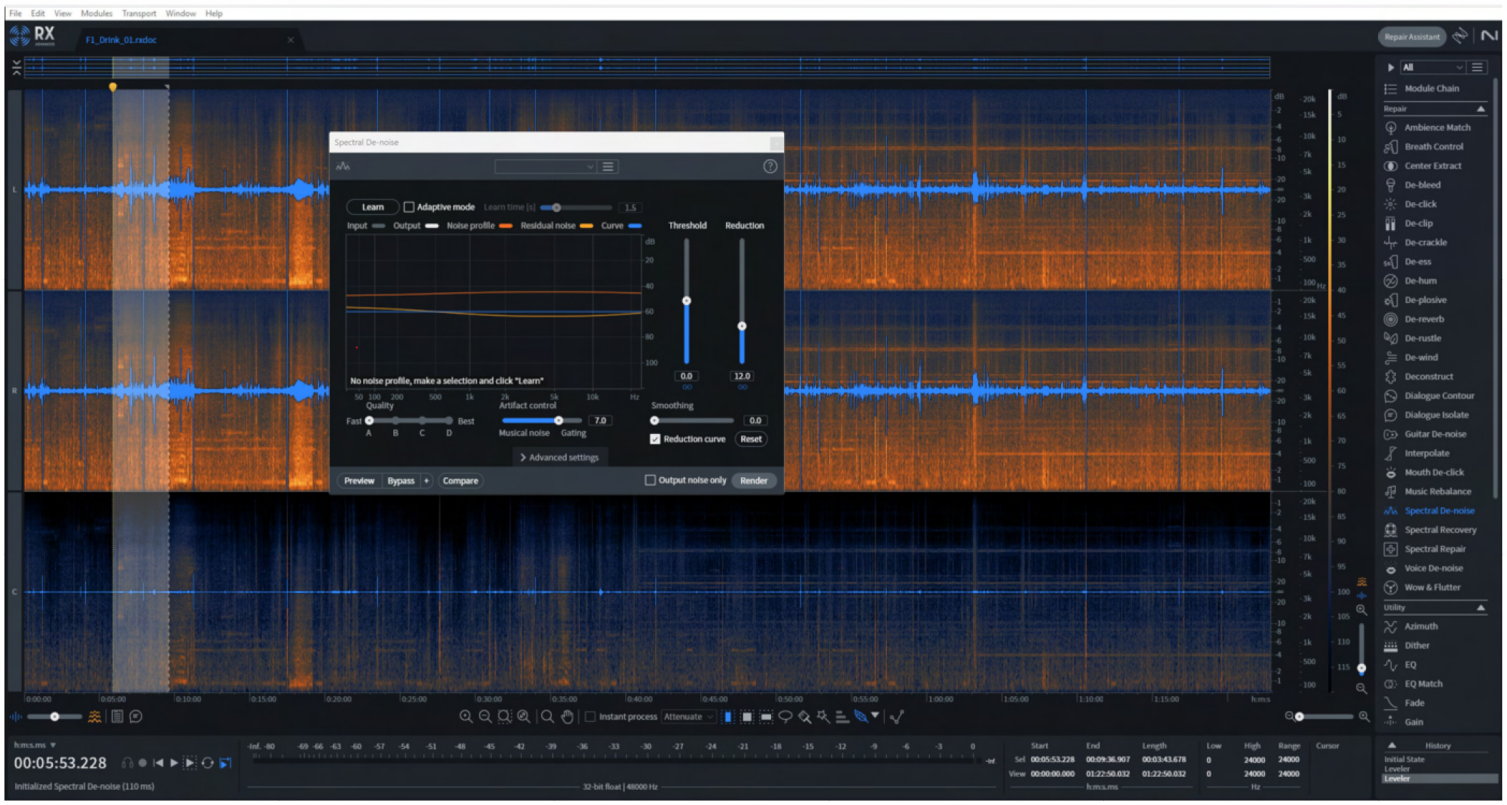
Spectral denoising of a cow vocalization in iZotope RX. Screenshot from iZotope RX (Spectral Denoise module) showing the noise-reduction workflow applied to raw barn audio prior to feature extraction. The **(upper panel)** displays the original spectrogram with broadband background noise typical of fan and machinery hum, while the **(lower panel)** shows the cleaned signal after adaptive spectral subtraction. Noise profile estimation was performed using a noise-only segment, and attenuation settings were tuned to preserve vocal harmonics while suppressing stationary low-frequency noise and high-frequency hiss. This preprocessing step enhanced signal-to-noise ratio and ensured clearer spectral features for subsequent analysis.

This pipeline produced a notable improvement in signal-to-noise ratio, such that cow vocalizations became more distinct in spectrograms, with cleaner harmonic continuity and fewer artifact interruptions. The enhanced clarity aids both manual annotation and downstream segmentation or feature extraction. A screenshot of the RX 11 interface showing a spectral editing session (Figure 6) visually demonstrates how noise is isolated and removed while maintaining vocal structure. Although the approach does not fully eliminate overlapping noise within the vocal band, it significantly reduces interference and lays the foundation for robust event detection and feature analysis.

Because iZotope RX 11 is proprietary software, we implemented a fully open-source Python pipeline that closely approximates this multi-stage workflow and can be executed without commercial tools. Starting from the band-pass filtered audio described in Section 4.2, the Python script performs:

1. **Gain normalization and DC-offset removal**: Audio is loaded, the mean is subtracted to remove DC offset, and levels are peak- or loudness-normalized to a consistent, non-clipping range.
2. **Broadband spectral de-noising**: Non-stationary spectral gating with noisereduce attenuates barn background noise, using noise-only segments when available or adaptive noise estimation otherwise, with moderate reduction strength to preserve vocal harmonics.
3. **Spectral repair of transient artifact**: Short broadband transients (e.g. metallic hits, clicks) are detected in the STFT domain librosa and locally attenuated or smoothed in the time-frequency representation.
4. **Optional de-clip and de-crackle**: Clipped peaks are reconstructed by interpolation between unclipped neighbors, and fine impulsive crackle is reduced using simple median-style filtering, applied only when clear recording faults are present.
5. **Spectral envelope / EQ matching**: Average magnitude spectra of each clip are matched to a neutral reference using a frequency-wise gain curve numpy, scipy.signal, reducing microphone- and placement-dependent coloration.

This five-step workflow provides a transparent, fully reproducible alternative to the RX 11 chain; although the Python implementation follows the same processing philosophy and sequence, individual output waveforms will not match RX 11 results sample-for-sample and may differ slightly in residual noise and timbre. The complete Python implementation and configuration details for this pipeline are available in the GitHub repository referenced in the Data Availability Statement.

### 4.4 Segmentation with Raven lite and acoustic inspection in Praat

After denoising in iZotope RX 11, we segmented the continuous recordings into individual vocalizations using Raven Lite (Version 2.0). Raven Lite is a free audio analysis tool developed by the Cornell Lab of Ornithology and widely applied in ecological and behavioral bioacoustic studies (Dugan et al., 2016). Raven Lite was selected because it provides real-time waveform and spectrogram visualization with straightforward selection tools for manual clipping and export, and it is freely available and widely used in bioacoustic workflows. Although it lacks automated detection and batch-processing features available in Raven Pro, it was well-suited for the context-aware, manual segmentation required in this study.

**Figure 7:**
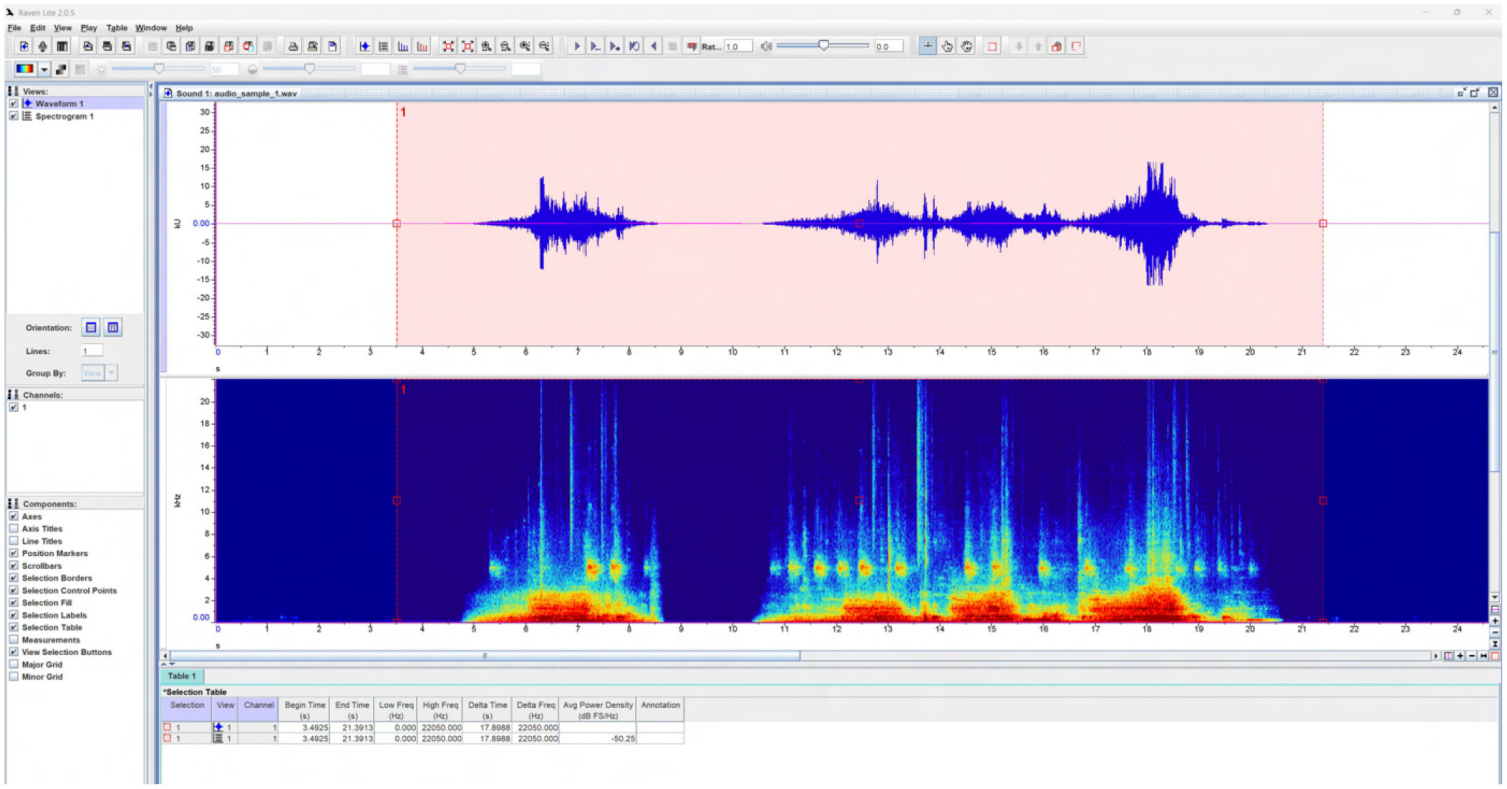
Annotation of cow vocalizations using Raven Lite. Screenshot from Raven Lite showing manual annotation of vocalization segments on the spectrogram view. Each selection box corresponds to an identified call event, with start and end times marked based on visible harmonic onset and offset boundaries. This visual verification ensured accurate segmentation of vocalizations and exclusion of mechanical or environmental noise. Annotated time-frequency regions were later used as reference intervals for automated feature extraction and labeling in Python.

We configured Raven Lite to display a short-time Fourier transform (STFT) spectrogram with a 1,024-point window, 50% overlap, and a Hamming window (Figure 7) which strikes a practical balance between time and frequency resolution for cattle calls. Candidate events were identified by visually scanning the spectrogram for harmonic stacks or broadband bursts and confirming each event by listening. Each clip was then extracted with start-end markers, and 2–3 s of padding were included on either side to preserve contextual cues (e.g., pre-onset inhalation, resonance tails). Onset was marked where amplitude rose above the noise floor and the first harmonic band became visible; offset was marked where energy returned to baseline–criteria consistent with common bioacoustic segmentation practice in livestock vocal studies (Meen et al., 2015).

To maintain traceability, each selection was exported as a new WAV file and named with a structured convention encoding farm, zone, date, time, microphone, and a provisional class placeholder. Approximately 569 clips were extracted from 90 h of recordings. To reduce subjectivity, an independent second annotator cross-checked a subset of clips for boundary placement and completeness (inter-observer calibration), and we adopted a conservative policy of retaining extra seconds of context when uncertain.

**Figure 8:**
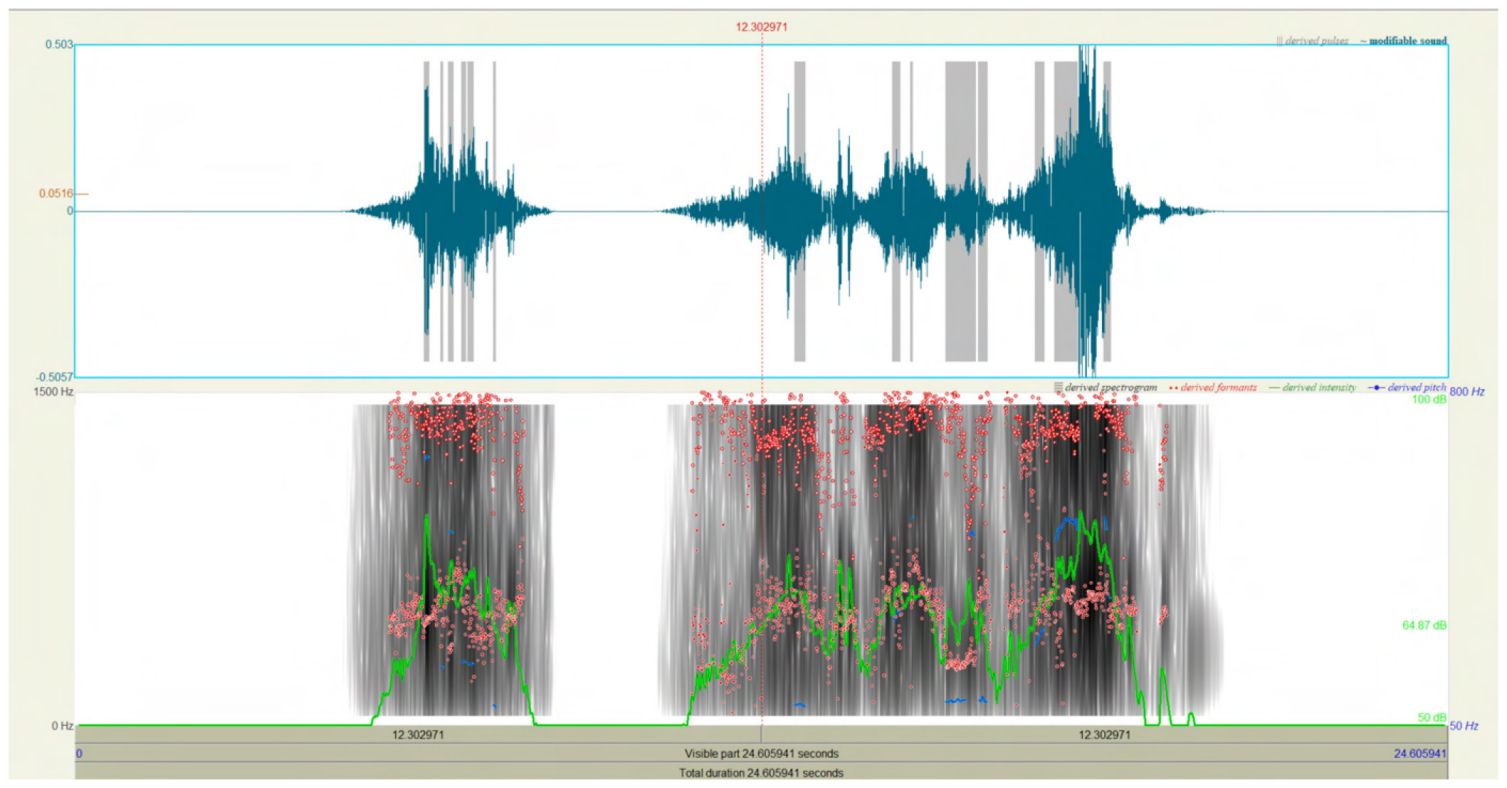
Acoustic inspection and feature verification in Praat. Screenshot from Praat showing waveform, spectrogram, pitch contour (F_0_), formant tracks (F_1_-F_2_), and intensity envelope for a representative cow vocalization. Visual inspection in Praat was used to verify segmentation boundaries and confirm that selections represented bona fide vocal events rather than background transients. Continuous harmonic structure and expected formant trajectories were checked against known patterns of bovine vocal production, ensuring that annotated clips reflected valid call behavior prior to automated feature extraction and modeling.

Following clipping, we performed acoustic inspection in Praat (Boersma, 2011) to verify segmentation boundaries and confirm that selections represented bona fide vocalizations rather than residual noise or mechanical transients (Green et al., 2019). In Praat (Figure 8), we inspected pitch contours (F0) and formant tracks (F1, F2) alongside intensity envelopes to check that harmonic structure was continuous within marked boundaries and aligned with cattle-vocal production expectations reported in prior work. We then bridged Praat to Python via Parselmouth (Jadoul et al., 2018) for scripted extraction of Praat-native measures (e.g., pitch range, formants, harmonic-to-noise ratio), and complemented these with librosa features (e.g., spectral centroid, bandwidth, roll-off, zero-crossing rate) for machine-readable descriptors used later in modeling. This combination ensured consistency between human-verified boundaries and algorithmic features.

Raven Lite's strengths at this stage are its stability, clear spectrogram interface, and low operational overhead for manual, context-aware segmentation; limitations include the lack of batch annotation and advanced detectors, which exist in Raven Pro and in research code. As the dataset expands, we anticipate scaling via semi-automated detectors (e.g., HMM/ML pipelines or Raven Pro's detection tooling) as demonstrated in related cattle-vocal detection studies. For the present work, however, manual segmentation with conservative padding and cross-validation produced a high-confidence corpus appropriate for downstream feature extraction and analysis.

To quantify how consistently this annotation protocol could be applied by independent observers, we conducted an inter-annotator agreement study on a stratified subset of clips, as described in Section 4.5.

### 4.5 Inter-annotator agreement and reliability

To assess the reliability of the behavioral and acoustic labels applied to the curated clips, we conducted an inter-annotator agreement study on a stratified subset of 150 audio clips. The subset was sampled to cover all nine main behavioral categories used in this study and the full set of 48 sub-categories, ensuring representation across diverse vocal types and barn contexts. Each clip in this subset was independently annotated by two trained observers following the same guidelines as described in Section 4.6, including assignment of a main category, sub-category, and associated contextual notes.

Agreement statistics were computed in Python using pandas and the cohen_kappa_score function from scikit-learn for nominal labels. A summary of inter-annotator reliability for both main and sub-category labels is provided in Table 5. The For the main behavioral categories (9 classes), direct comparison between Annotator 1 and Annotator 2 yielded a Cohen's κ of 0.884 with 90.0% raw agreement across the 150 clips, indicating high consistency in assigning broad behavioral classes. For the more fine-grained sub-categories (48 classes), inter-rater agreement remained substantial, with κ = 0.699 and 71.3% raw agreement.

**Table 5:** Inter-annotator agreement for main and sub-category labels (150 clips).

**Comparison**	**Label level**	**Clips**	**Cohen's κ**	**Percent agreement**
Annotator 1 vs Annotator 2	Main category (9 classes)	150	0.884	90.0%
Annotator 1 vs Annotator 2	Sub-category (48 classes)	150	0.699	71.3%
Final labels vs Annotator 1	Main category	150	0.930	94.0%
Final labels vs Annotator 2	Main category	150	0.954	96.0%
Final labels vs Annotator 1	Sub-category	150	0.832	84.0%
Final labels vs Annotator 2	Sub-category	150	0.860	86.7%

We also evaluated agreement between each annotator and the final consensus labels, which represent the adjudicated “gold-standard” annotations used in the released dataset. Consensus labels (“Main Category final” and “Sub Category final”) were derived through joint review by both annotators and the lead investigator, with reference to spectrograms, waveform envelopes, metadata, and co-recorded video where available. For main categories, agreement between the final labels and Annotator 1 was κ = 0.930 (94.0% agreement), and between the final labels and Annotator 2 was κ = 0.954 (96.0% agreement). The top confusing pairs are shown in Supplementary Figures S1, S2. These reliability outcomes confirm that the finalized labels provide a stable ground truth for subsequent first-level annotation and downstream behavioral analyses.

### 4.6 First level annotation

Each segmented clip was subjected to a first-level annotation by two researchers, who assigned multiple labels reflecting call type, emotional context, and confidence, as well as a behavioral summary. Specifically, for each clip the annotator recorded:

- The main category and subcategory according to the scheme defined in Section 2.4, grounded in ethological typologies of vocalizing behavior;
- An emotional context label (distress, pain, anticipation, hunger), reflecting the putative affective state at the time of vocalization;
- A confidence score (1 = low to 10 = high) indicating the annotator's certainty in their labeling;
- A textual description summarizing observable behavioral cues or situational context (e.g. “calf standing near water trough,” “cow waiting at feed gate,” “walking past milking parlor”).

Onset and offset boundaries were further refined within Praat to exploit its high-precision time measurement and pitch/formant display capabilities, allowing annotators to fine-tune temporal limits of calls. The annotation guidelines drew from ethological principles: calls were to be linked to proximate stimuli (e.g. feeding, separation, disturbance) and considered in light of possible ultimate functions (e.g. contact, distress, solicitation). In ambiguous cases or overlapping calls, the label “Unknown” was assigned for later review rather than forcing a classification. Annotators also consulted manual notes and co-recorded video when available to confirm behavioral context (for instance, verifying whether a cow was feeding vs. showing frustration). The output of this stage is a curated set of labeled audio clips, each with category, emotional valence, confidence, and behavioral description, ready for downstream feature extraction and statistical modeling.

In determining the type of call, annotators relied on the spectro-temporal shape (e.g. harmonic stacks, frequency modulation, call duration), amplitude envelope, and context. For example, low-frequency calls with stable harmonics might be assigned to contact or low-arousal categories, whereas calls with abrupt onset, rapid modulation, or high spectral energy could indicate agitation or alarm calls. This practice aligns with literature on cattle vocalization as communicative signals bearing information about motivation or affect (Green et al., 2018) and more broadly on vocal behavior as an “ethotransmitter” in mammals (Brudzynski, 2013). In recent machine-learning work (e.g. Gavojdian et al., 2024, “BovineTalk”), cattle calls are classified into low-frequency (LF) and high-frequency (HF) types—often mapping LF to close-contact or neutral states and HF to more urgent or negative states—which lends empirical precedent to our annotation categories.

In summary, the preprocessing pipeline we employed—spanning quantitative noise profiling, targeted filtering, professional denoising, careful segmentation, and this rigorous, context-aware annotation—addresses the challenges of noisy barn environments and produces a high-quality, well-documented corpus of vocalizations. This corpus forms a robust foundation for feature extraction and subsequent machine learning or statistical modeling. After preprocessing and initial annotation, we proceeded to compile the dataset through feature extraction, biological interpretation, metadata compilation, and exploratory analysis.

## 5 Dataset creation

### 5.1 Acoustic feature extraction

Once the segmented clips had undergone first-level annotation, we extracted a comprehensive suite of 24 acoustic features to characterize each vocalization, as summarized in Table 6. Feature computation was carried out using a combination of Praat, Parselmouth, librosa, and openSMILE, representing both traditional phonetic tools and modern signal-processing libraries. The features spanned temporal, spectral, and cepstral domains, with the aim of capturing both biologically interpretable measures and machine-readable descriptors commonly employed in animal bioacoustic research (Meen et al., 2015; de la Torre et al., 2015).

- **Temporal metrics** included onset time, offset time, and call duration. Duration has been repeatedly linked to arousal and motivational state: shorter calls are more often associated with neutral or positive contexts, while longer calls typically reflect higher arousal or negative states (Brudzynski, 2013).
- Signal quality was quantified using **signal-to-noise ratio** (SNR), allowing assessment of how clearly the vocalization emerged from the barn environment. High-SNR clips ensure more reliable feature measurement and downstream modeling.
- **Fundamental frequency** (F0) was measured using Praat's autocorrelation method, with statistics for mean, minimum, and maximum F0 calculated for each clip. Cattle vocalizations generally fall between 50 and 1,250 Hz, with typical averages of 120–180 Hz. High-frequency calls near 150 Hz have been linked to separation distress, whereas low-frequency nasal calls around 80 Hz are associated with close contact and calming social functions (Watts and Stookey, 2000; Green et al., 2018; Gavojdian et al., 2024).
- **Intensity** statistics (minimum and maximum dB) captured variation between quiet and forceful calls. In line with previous findings, higher-intensity calls often indicate urgency or frustration, while lower intensities correspond to calm affiliative contexts (Watts and Stookey, 2000).
- **Formant frequencies** (F1, F2) were estimated using Praat's Burg method. Formants represent resonances of the vocal tract and provide cues to body size and vocal tract shape (Fitch and Hauser, 2003). In cattle, F1 and F2 have been documented in ranges between ~ 228 and 3,181 Hz, with average call durations of ~ 1.2 s (de la Torre et al., 2015).
- **Band-level metrics** were extracted using librosa, including bandwidth, RMS energy (mean and standard deviation), spectral centroid, spectral bandwidth, spectral roll-off (85/95), zero-crossing rate (ZCR, mean and standard deviation), and time to peak energy. These descriptors summarize the energy distribution and noisiness of the call. For example, high spectral centroid values correspond to brighter, noisier events (e.g., metallic barn sounds, snorts), whereas lower centroid values are characteristic of harmonic moos.
- **Mel-frequency cepstral coefficients** (MFCCs) (first 13 coefficients: mean and standard deviation) were computed using librosa and openSMILE. MFCCs provide a compact representation of vocal timbre and are widely used in classification of animal calls, including cattle vocalizations (Schrader and Hammerschmidt, 1997; Sattar, 2022).
- Finally, the **voiced rati**o was calculated, representing the proportion of frames classified as voiced vs. unvoiced. High-frequency distress calls often exhibit more unvoiced frames due to glottal widening and turbulent airflow, while nasal low-frequency moos are typically fully voiced (Briefer, 2012).

**Table 6:** Acoustic feature set extracted from segmented cattle vocalizations.

**Feature**	**Description**	**Extraction tool / method**
Start time, end time, duration	Temporal metrics providing call timing and length. Duration has been linked to arousal and behavioral state: shorter calls often reflect neutral/positive states, longer calls higher arousal.	Praat / parselmouth
Signal-to-Noise Ratio (SNR)	Measures clarity of the call relative to barn noise; ensures robust downstream feature reliability.	Custom script (waveform-based)
Fundamental frequency (F0: mean, min, max)	Pitch contour statistics; cattle vocal range ~50–1250 Hz. High frequencies linked to distress/separation, low nasal calls (~80 Hz) to close contact.	Praat autocorrelation / Parselmouth
Intensity (min, max, mean dB)	Energy levels of the vocalization; high intensity = urgency/frustration, low intensity = calm contact.	Praat / Parselmouth
Formants (F1, F2)	Resonant vocal tract frequencies; provide cues to body size and vocal configuration.	Praat Burg method
Spectral centroid	Center of mass of spectrum; high values = bright/noisy, low values = harmonic moos.	librosa
Spectral bandwidth	Spread of spectral energy around the centroid.	librosa
Spectral Roll-off (85%, 95%)	Frequency below which 85% or 95% of spectral energy is contained.	librosa
Zero crossing rate (mean, std)	Rate of waveform sign changes; indicates noisiness/aperiodicity.	librosa
RMS energy (mean, std)	Root mean square energy; reflects call strength and stability.	librosa
Time to peak energy	Time taken for a call to reach maximum energy; a dynamic marker of urgency.	librosa / scipy
Mel Frequency Cepstral Coefficients (MFCCs 1–13: mean, std)	Cepstral features capturing vocal timbre; widely used in vocal classification.	librosa / openSMILE
Voiced ratio	Proportion of voiced vs. unvoiced frames; distress calls often more voiceless, nasal moos nearly fully voiced.	Praat / Parselmouth

All features were extracted at a sampling rate of 16 kHz (down-sampled from 44.1/48 kHz), which preserved the relevant bovine vocal bandwidth while reducing computational load.

Beyond numerical computation, these features hold biological meaning, which we interpret in the next subsection.

### 5.2 Biological interpretation of acoustic features

The selected features were chosen not only for their statistical utility in machine learning, but also for their biological interpretability. This dual emphasis ensures that computational models remain grounded in the mechanisms of cattle vocal production and their ethological significance.

- **Fundamental frequency** (F0) is primarily determined by the length, tension, and mass of the vocal folds (Brudzynski, 2010). Increases in F0 are commonly associated with heightened arousal, separation distress, or estrus, while lower F0 is characteristic of calm affiliative calls (Green et al., 2018; Röttgen et al., 2018). In our dataset, calls labeled Estrus_Call and High_Frequency_Distress exhibited both elevated maximum F0 and greater F0 variability, consistent with reports that female cattle emit higher-pitched calls during estrus or when separated from their calves. Conversely, low-frequency nasal moos, typically around 80-120 Hz, were more often associated with affiliative or contact-seeking contexts, echoing findings from maternal-offspring communication studies (de la Torre et al., 2015).
- **Formant frequencies** (F1, F2) reflect vocal tract resonances and convey information about articulatory configuration and body size (Fitch and Hauser, 2003). Lower formant values are linked to mouth opening and longer vocal tract length, while higher formants reflect tongue and lip positioning. In our data, Water_Slurping_Sounds displayed broad bandwidths and high F1-F2 separation, capturing their noisy, non-harmonic structure, whereas harmonic moos showed tighter clustering of F1 and F2 bands. This observation is consistent with earlier descriptions of formant dynamics in cattle vocalizations (Watts and Stookey, 2000).
- **Energy-based measures** (RMS energy, intensity, and time to peak) further captured the dynamic and affective force of vocalizations. High RMS energy and short time-to-peak values were prominent in Frustration_Calls and Aggressive_Bellows, reflecting abrupt, high-force emissions. By contrast, Mother_Separation_Calls were lower in intensity but longer in duration, representing persistent vocal efforts at lower force levels, in line with observations of separation calls in cow-calf pairs (Green et al., 2019).
- **Spectral descriptors** provided a robust means of distinguishing harmonic from noisy events. Spectral centroid and roll-off values differentiated stable harmonic moos from broadband, non-vocal sounds such as sneezes, coughs, or burps. These non-vocal events showed elevated centroid and zero-crossing rate values, indicating their noisy and aperiodic character (Meen et al., 2015).
- Finally, **Mel-frequency cepstral coefficients** (MFCCs) encoded global spectral shape and proved particularly useful for distinguishing subtle differences between similar categories such as Feed_Anticipation_Call and Feeding_Frustration_Call. This aligns with recent applications of MFCCs in livestock monitoring, where cepstral analysis has been shown to detect estrus events with accuracies exceeding 90% (Sattar, 2022; Gavojdian et al., 2024).

Taken together, these feature-behavior associations reinforce that the dataset is not only suitable for computational modeling, but also biologically meaningful. By anchoring machine-readable features to established ethological frameworks (Tinbergen's four questions; Dugatkin, 2020), the approach ensures that downstream classification and prediction retain relevance to animal welfare science and practical dairy farm monitoring.

To ensure each feature and annotation is transparent and reusable, we compiled a structured metadata schema.

### 5.3 Metadata compilation and schema

To ensure that each audio clip could be unambiguously identified, contextualized, and reused for further research, we developed a comprehensive metadata schema to accompany the curated dataset (Table 7). The schema integrates identifiers, contextual descriptors, acoustic parameters, behavioral annotations, and environmental measures, thereby aligning with best practices for animal genomics and behavioral data curation as outlined by the FAANG consortium (Harrison et al., 2018) and FAIR data principles.

**Table 7:** Metadata schema accompanying each segmented clip.

**Field**	**Description**	**Data type**	**Example**
File Name	Unique identifier for the clip, following structured naming convention	String	Farm1_Drinking_2025-05-05_10-45_Mic2_Feed_Anticipation.wav
Original File	Parent recording file name	String	Farm1_Day1_WaterStation.wav
Date	Date of recording	Date (YYYY–MM–DD)	2025-05-06
Time	Time of recording	Time (HH:MM:SS)	10:45:32
Farm ID	Unique identifier for farm	Integer / String	Farm1
Barn Zone	Location within barn (e.g., feeding, water, resting, milking area)	String	Feeding Zone
Microphone Model	Microphone model used	String	Rode NTG2
Recorder	Recorder type	String	Zoom H4n Pro
Mic Placement Context	Placement or mounting details	String	Above feed trough
Start Time (s)	Start time of clip within original recording	Float (seconds)	938.03
End Time (s)	End time of clip within original recording	Float (seconds)	963.84
Duration (s)	Call duration	Float (seconds)	25.81
Fundamental Frequency (F0)	Mean, min, and max pitch values	Float (Hz)	Mean: 150 Hz; Max: 310 Hz
Formants (F1, F2)	First and second formant frequencies	Float (Hz)	F1 = 320 Hz, F2 = 1180 Hz
Energy Metrics	Intensity (min, max, mean dB); RMS energy	Float (dB)	Min = 55 dB, Max = 70 dB
Spectral Features	Centroid, bandwidth, roll-off, zero crossing rate, time to peak	Float / Derived	Centroid = 1120 Hz; ZCR = 0.08
MFCCs (1–13)	Mean and standard deviation of MFCC coefficients	Array (floats)	[12.4, 9.6, …]
Voiced Ratio	Proportion of frames classified as voiced vs. unvoiced	Float (%)	92% voiced
Main Category	Behavioral category (from annotation scheme)	String	Feeding and Hunger Related
Sub Category	Specific vocal type	String	Feed_Anticipation_Call
Emotional Context	Affective state inferred from annotation	String	Positive
Confidence Score	Annotator certainty level (1 = low, 3 = high)	Integer (1–3)	3
Description	Free-text summary of behavior/context	String	Cow waiting at feed gate
Low Frequency Bound	Lower cutoff of applied band-pass filter	Float (Hz)	50 Hz
High Frequency Bound	Upper cutoff of applied band-pass filter	Float (Hz)	1800 Hz
Bandwidth	Effective bandwidth of call	Float (Hz)	1750 Hz
Signal-to-Noise Ratio	Call clarity relative to background	Float (dB)	15.3 dB

Metadata fields were structured into five categories:

1. **File identifiers:** Each clip is assigned a unique filename following a structured convention that encodes farm ID, recording zone, date, time, microphone ID, and provisional call type. Additional fields include the original recording filename and precise date-time stamps.
2. **Contextual information:** These fields capture the recording environment and instrumentation, including farm identifier, barn zone (e.g., feeding area, water station, resting area, milking parlor), microphone model, recorder type, and mic placement context. Such metadata ensure interpretability across heterogeneous barn environments (Meen et al., 2015).
3. **Acoustic features:** The full set of 24 acoustic features described in Section 5.1—including duration, F0 statistics, formant values, intensity measures, spectral centroid, bandwidth, roll-off, RMS energy, zero-crossing rate, MFCC statistics, voiced ratio, and time-to-peak energy—are included. Storing these features directly in the metadata table enables rapid subsetting and analysis without rerunning extraction pipelines.
4. **Behavioral annotations:** Each clip is linked to the annotation schema described in Section 4.6. Fields include the main category and subcategory (e.g., Feeding_Anticipation_Call, Mother_Separation_Call, Burp), emotional context (discomfort, pain, hunger, thirst), annotator confidence score (1-10), and free-text description summarizing behavioral cues (e.g., “calf standing near water trough,” “cow waiting at feed gate”). This dual annotation—structured categories plus free-text notes—enables both quantitative and qualitative analysis.
5. **Environmental parameters:** To document recording conditions and preprocessing steps, metadata also include the low and high frequency cut-offs from the band-pass filter (Section 4.2), effective bandwidth, signal-to-noise ratio, and microphone gain settings where available. These parameters are critical for reproducibility, given the variability of barn acoustic environments (Alsina-Pagès et al., 2021).

The metadata schema thus supports Findable, Accessible, Interoperable, and Reusable (FAIR) data practices by providing clear identifiers, structured descriptors, and machine-readable acoustic metrics. To illustrate, Tables 8, 9 presents three representative metadata records: a Feed_Anticipation_Call recorded in the feeding zone, a Mother_Separation_Call in the resting area, and a Burp in the feeding zone. These examples demonstrate how behavioral categories, recording contexts, and acoustic profiles (e.g., differences in duration, F0 maxima, intensity, and formant dispersion) are transparently documented.

**Table 8:** Rep. metadata records (A): identifiers and context.

**File name**	**Barn zone**	**Category**	**Description**
Feeding_Hunger_Empty_Feeder_Call_34s	Feeding	Empty_Feeder_Call	Complaint at empty feeder
Maternal_Calf_Separation_Mothercow_Call_13s	Resting	Mother_Separation_Call	Loud, urgent call for calf
Non_Vocal_Chewing_Burping_Sound_07s	Feeding	Burp	Chewing and cud sounds

**Table 9:** Rep. metadata records (B): acoustic measurements.

**Duration (s)**	**F0 Max (Hz)**	**Intensity (dB)**	**Formant 1 (Hz)**	**Formant 2 (Hz)**	**File name (short)**
34.22	368.0	26.65	650.67	1653.99	Empty_Feeder_Call_34s
13.00	615.3	60.44	1079.64	1913.06	Mother_Separation_Call
6.84	29.7	28.89	740.71	1951.26	Burping_Sound_07s

By compiling both biological and technical descriptors, the metadata not only enable efficient subsetting of the corpus (e.g., by behavior, environment, or acoustic property), but also ensure long-term reproducibility and interoperability with other livestock bioacoustic datasets.

### 5.4 Class distribution, augmentation, and class balancing

**Figure 9:**
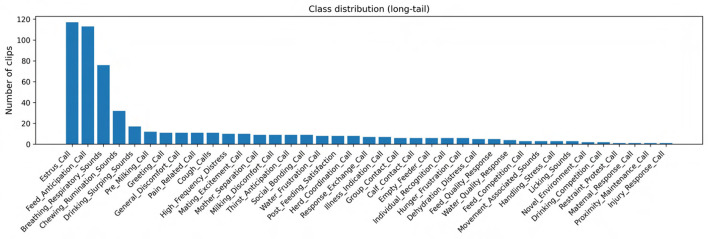
Overall class distribution of cow vocalizations. Long-tail distribution of all annotated classes in the dataset. Each bar represents the number of audio clips within a behavioral or acoustic category. A pronounced imbalance is evident, with a few highly represented classes (e.g., estrus and anticipation calls) and many rare categories such as response exchange or distress calls, reflecting the natural skew of on-farm acoustic events.

**Figure 10:**
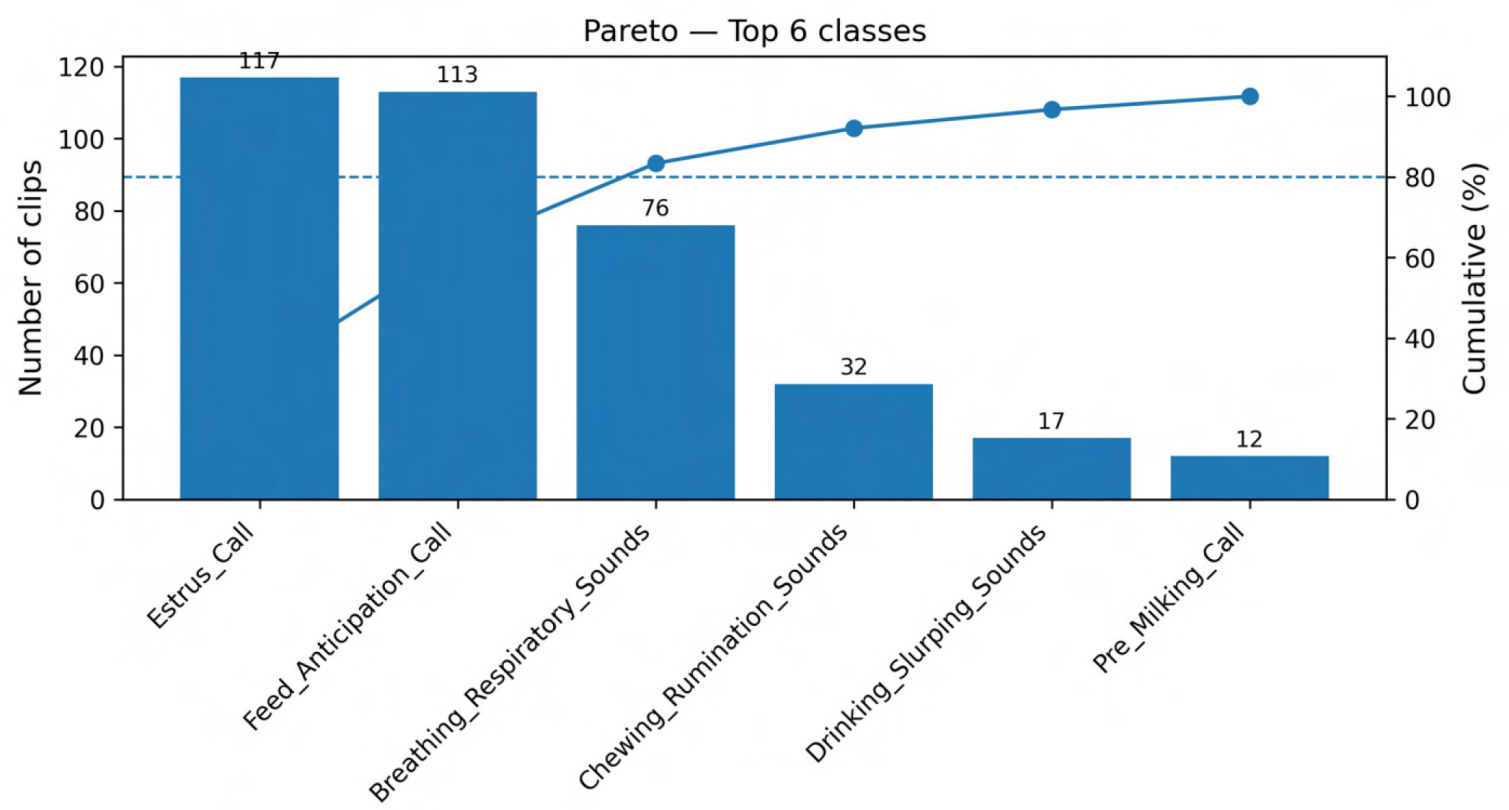
Pareto chart of top six vocalization classes. Distribution of the most frequent vocalization categories in the dataset, displayed as a Pareto chart. Bars indicate the number of clips per class, and the overlaid line shows the cumulative proportion of all clips. The six dominant classes together account for the majority of recorded samples, illustrating the long-tailed nature of class occurrence and guiding model evaluation toward balanced and minority-aware analysis.

The final curated dataset comprises 569 audio clips spanning 48 behavioral classes. Each clip is a denoised vocalization or nonvocal sound with precise onset and offset boundaries. The mean duration is ~ 21 s (median ~ 13.8 s; range 2.8-445 s), with ~ 75 % of clips shorter than 21 s. The raw class distribution is strongly long-tailed: a small number of behavior types (e.g., estrus calls, feed anticipation calls, and respiratory sounds) account for a large fraction of clips, while many classes are represented by fewer than 10 examples. This pattern reflects both the true frequency of behaviors on farm and the practical difficulty of capturing rare events. The long-tailed distribution is illustrated by the histogram and Pareto plots in Figures 9, 10. Compared with earlier bovine vocalization datasets [e.g., 1,144 calls from 20 isolated cows (Gavojdian et al., 2024), or 290 calls across four physiological states (Yoshihara and Oya, 2021)], our corpus is broader in scope, encompassing multiple farms, barn zones, high- and low-frequency calls, and nonvocal events. Skewed distributions are common in animal bioacoustics (Kahl et al., 2021) and present well-documented challenges for machine learning models, which may overfit to frequent classes and underperform on rare but ethologically meaningful categories (Johnson and Khoshgoftaar, 2019).

To reduce this imbalance for model training, we implemented a simple but explicit class-balancing scheme applied only to the training set. First, we defined a target threshold of 81 clips per class. This value is close to the size of mid-frequency classes in the raw data and provides a practical upper bound that prevents a few very common behaviors from dominating the training regime. Classes with more than 81 original clips were subjected to majority undersampling. For these high-frequency classes, we randomly selected 81 clips without replacement to retain in the training set. For example, the Estrus Call and Feed Anticipation Call categories were reduced from their original counts to 81 training clips each. This down-sampling step limits the influence of extremely common behaviors while still preserving a diverse set of exemplars.

Classes with fewer than 81 clips but at least three original examples were then expanded via minority-class augmentation. For these categories, we generated additional clips using a small set of biologically plausible perturbations:

1. Time-stretching [(0.8–1.2×) of the original duration]
2. Pitch shifting by up to ±2 semitones,
3. Addition of low-level Gaussian noise (SNR ≥ 20 dB) to simulate varying background conditions.
4. Moderate gain adjustment (on the order of ±6 dB) to mimic changes in caller distance and vocal effort.

These augmentations simulate natural variability in vocal production and recording context, such as differences in caller distance, vocal effort, or microphone orientation. Importantly, augmentation parameters were constrained to remain within biologically plausible ranges: pitch shifts avoided unrealistic F0 values, while time-stretching was limited to ±20% to preserve temporal dynamics of vocal events (Briefer, 2012; Ganchev et al., 2005).

Extremely rare classes with fewer than three original clips were excluded from model training but retained in the metadata and dataset documentation. These behaviors (e.g., certain rare maternal or social calls) are clearly flagged as “Dropped” for training in our tables but remain part of the annotated corpus for transparency and for potential future aggregation with additional data.

After applying majority undersampling to very common classes and augmentation to under-represented ones, the training set expanded from 569 to approximately 2,900 clips, with a much more even per-class distribution. Importantly, this balancing procedure was applied only to the training split. The validation and test sets are composed exclusively of original, non-augmented clips, stratified by class, so that performance estimates reflect how models generalize to real recordings rather than synthetic variants. Table 10 presents the number of clips per class before and after augmentation, demonstrating how augmentation improved distributional balance.

**Table 10:** Representative clip counts before and after class balancing in the training set.

**Class**	**Before augmentation**	**After augmentation**
Estrus_Call	117	81
Feed_Anticipation_Call	113	81
Breathing_Respiratory_Sounds	76	81
Chewing_Rumination_Sounds	32	81
Drinking_Slurping_Sounds	17	81
Pre_Milking_Call	12	81
Cough_Calls	11	81
Pain_Related_Call	11	81
Maternal_Response_Call	1	Dropped
Restraint_Protest_Call	1	Dropped
Proximity_Maintenance_Call	1	Dropped

“Before augmentation” gives the number of manually segmented, denoised clips per behavioral subcategory; “After augmentation” shows counts after applying majority undersampling (for high-frequency classes) and synthetic augmentation (for under-represented classes with ≥3 clips). Classes with fewer than three clips were excluded from training (“Dropped”). Validation and test sets use only original, non-augmented clips.

By combining augmentation and balanced sampling, the approach reduced class bias and improved the capacity of models to generalize across both frequent calls (e.g., feeding anticipation, respiratory sounds) and rare but behaviorally significant calls (e.g., social recognition, separation). This methodology reflects best practice in animal bioacoustics, where careful augmentation helps capture the ecological variability of vocal signals without compromising their biological validity.

### 5.5 Preliminary feature analysis

**Figure 11:**
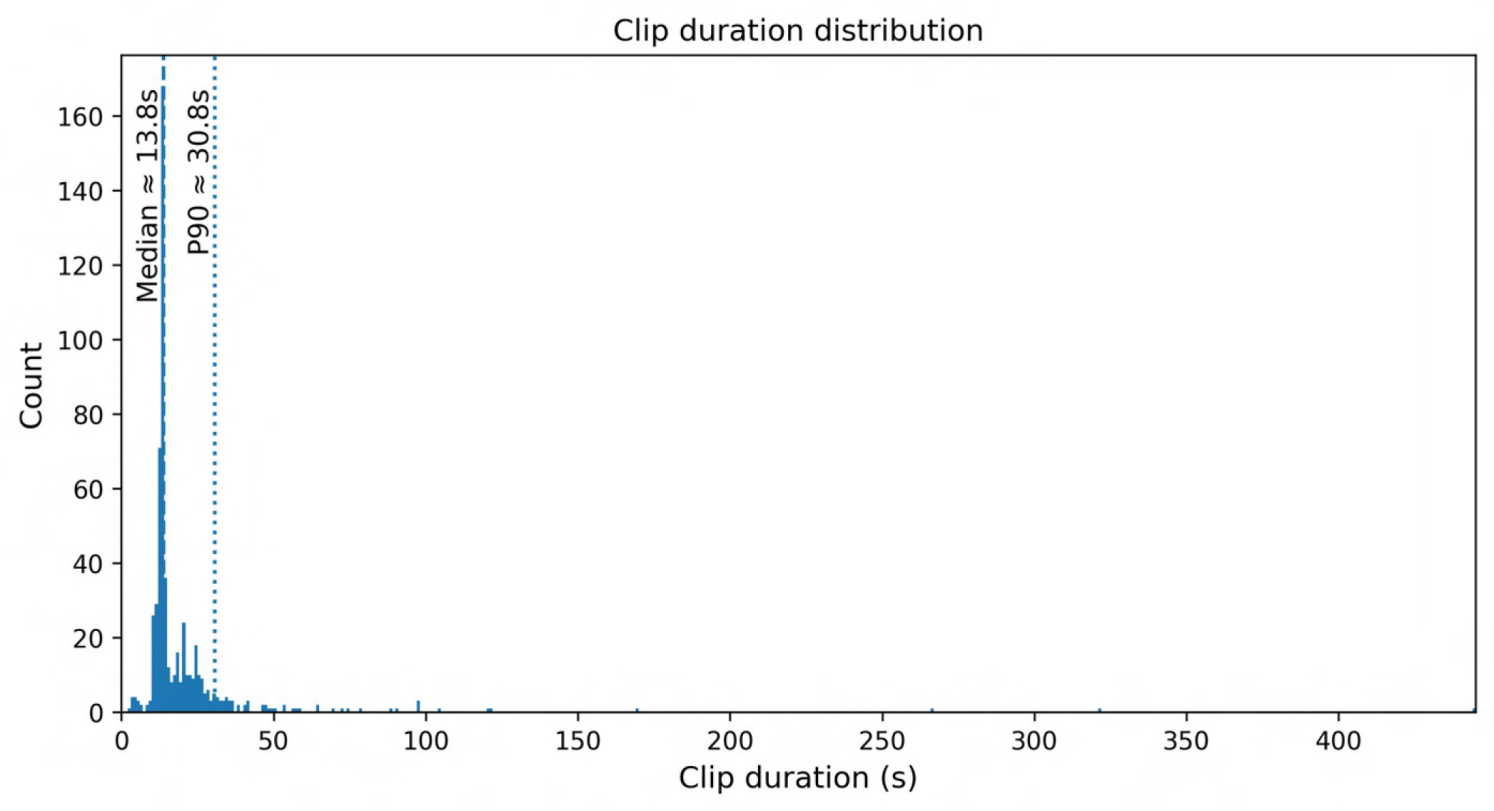
Clip duration distribution across the dataset. Histogram showing the temporal distribution of clip lengths for all annotated vocalizations. Most clips are short (under 20 s), while a small number extend beyond one minute, producing a heavy-tailed pattern. Dashed and dotted lines mark the median and 90th-percentile durations, respectively, highlighting the variability in call length across behavioral contexts.

To assess the discriminative power of the extracted features, we conducted exploratory analyses of temporal, spectral, and energy-based parameters across the dataset. Clip duration (Figure 11) exhibited a heavy-tailed distribution: the majority of clips were shorter than 20 s, although some extended beyond 200 s; the 90th percentile was ~60 s. This skewed distribution reflects the behavioral variability of cattle vocalizations, where most calls are short, context-bound signals, while prolonged calls occur during high-arousal contexts such as estrus or maternal separation (Röttgen et al., 2018; Green et al., 2018).

Pitch statistics (*F*_0_) (Figure 13) varied significantly across call categories. Non-vocal sounds like Breathing, Chewing, and Mother_Separation_Calls showed low mean *F*_0_ values (~100 Hz), consistent with affiliative and low arousal. In contrast, high-arousal vocalizations such as Estrus Calls, Feed Anticipation Calls, Greeting Calls, and Pre-Milking Calls clustered at substantially higher median *F*_0_ values (~415–425 Hz), while Cough Calls reached even higher medians (~440 Hz). These patterns are consistent with previous work linking elevated *F*_0_ to heightened arousal and distress in cattle and other mammals (Harrison et al., 2018; de la Torre et al., 2015), and qualitatively match the separation between low- and high-arousal classes visible in the violin plots.

**Figure 12:**
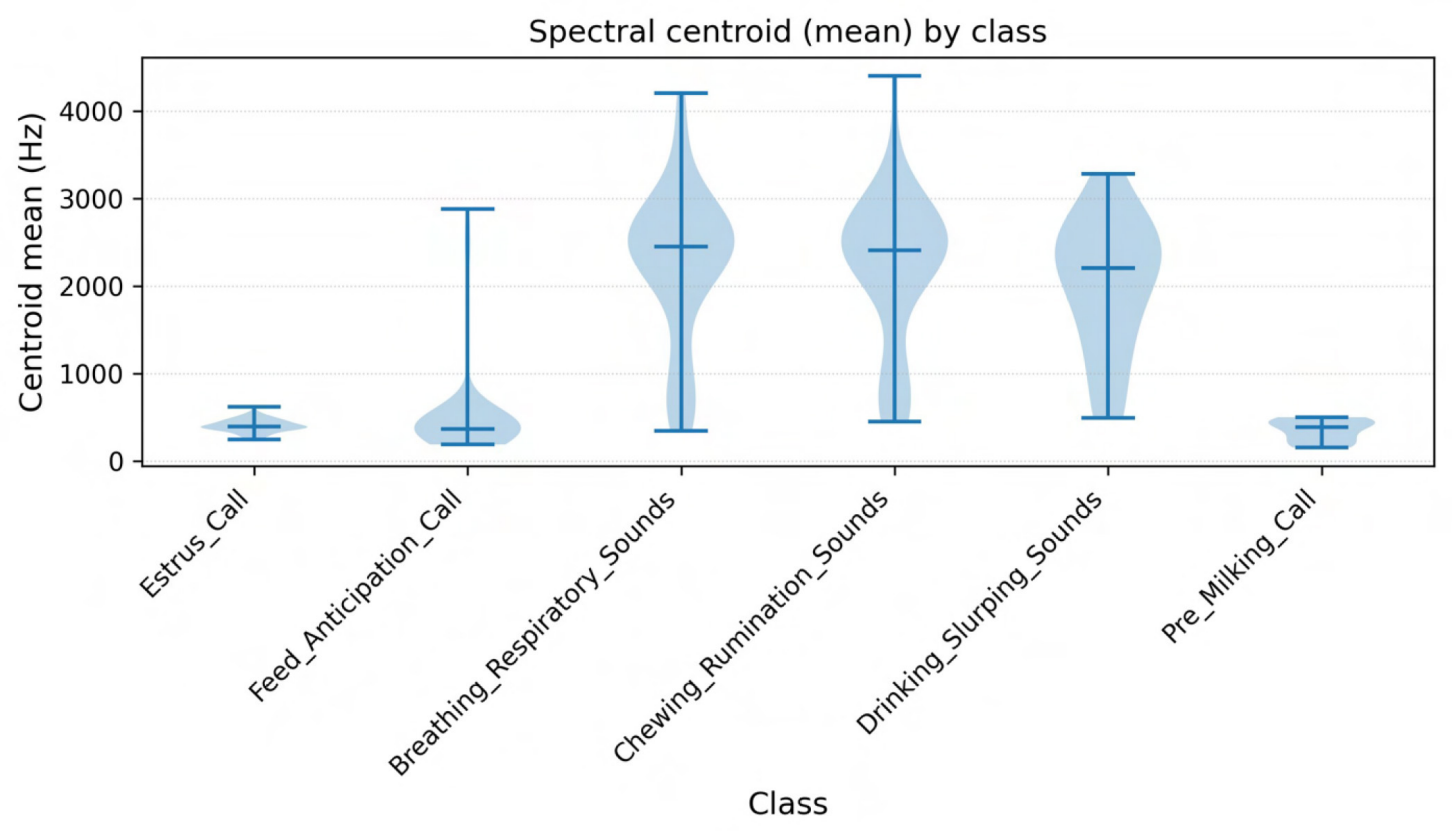
Violin plot of spectral centroid (mean) by class. Distribution of mean spectral centroid values for the top six classes. The spectral centroid describes the “brightness” of a sound, with higher values indicating stronger high-frequency components. The plot shows clear class-wise differences in spectral coloration–harmonic moos cluster at lower centroids, whereas short impulsive events (e.g., coughs, sneezes) show broader, higher-frequency spectra.

Energy and spectral measures (Figures 12, 14) also showed clear class-specific signatures. Non-vocal expulsive sounds such as Breathing Respiratory Sounds and Chewing Rumination Sounds were characterized by very high spectral centroid values (median ≈ 2.4 kHz), reflecting their broadband, noisy structure, and by relatively high RMS energy compared with many harmonic contact calls. Tonal vocalizations such as Estrus and Feed Anticipation Calls, as well as Pre-Milking Calls, instead clustered at much lower spectral centroids (medians ~370–400 Hz) with more concentrated spectral energy. RMS energy further differentiated classes: quiet, low-intensity events such as Pre-Milking and Chewing Rumination Calls tended to have low median RMS values (~−64 to −60 dB), whereas high-arousal Mating Excitement Calls and High Frequency Distress Calls were among the loudest signals (medians ~−22.5 dB and ~−29.9 dB, respectively), consistent with increased vocal effort during intense affective states (Meen et al., 2015).

**Figure 13:**
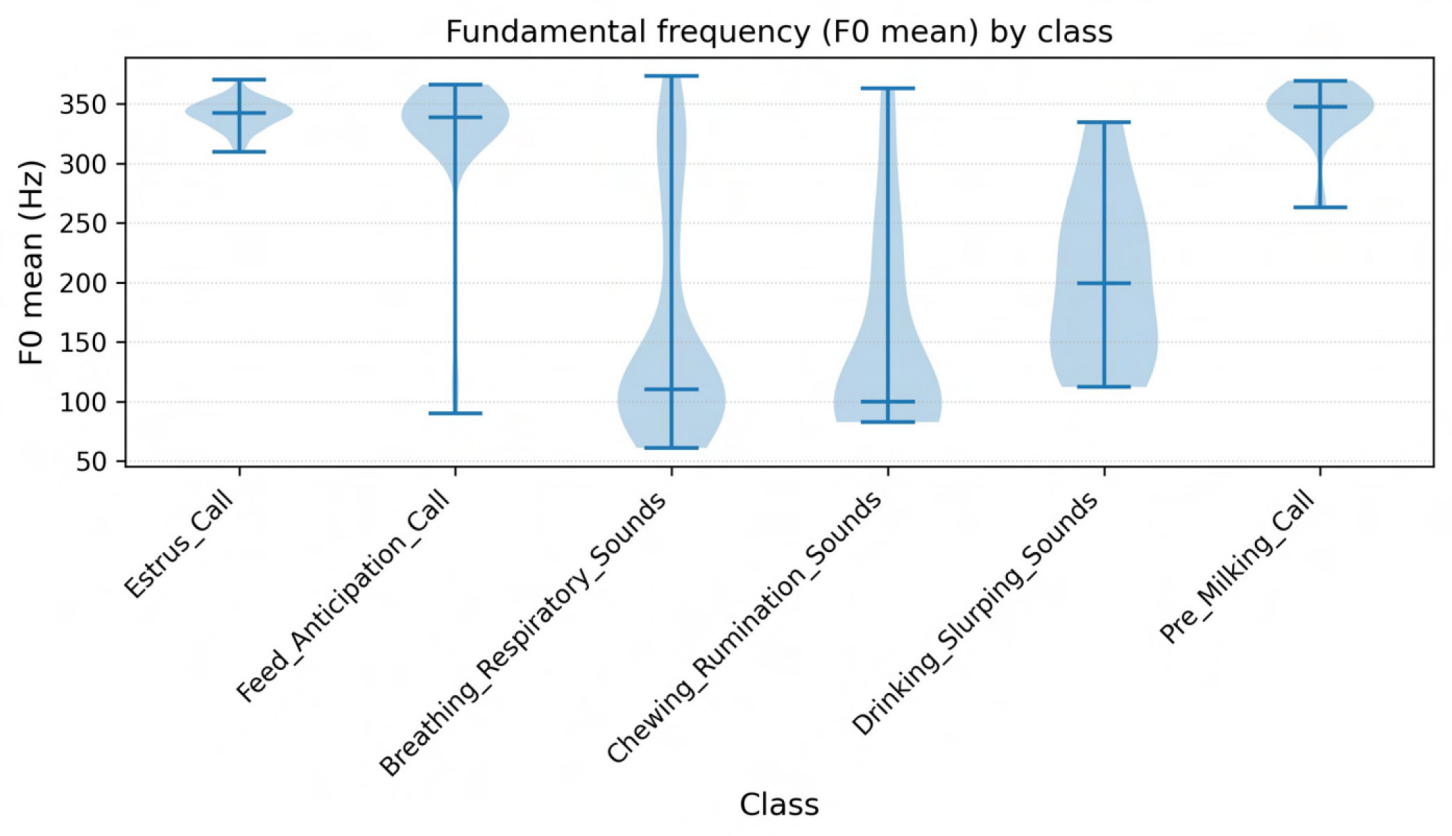
Violin plot of fundamental frequency (F_0_ mean) by class. Variation in mean fundamental frequency (F_0_) across the top six classes. Each violin depicts the distribution of F_0_ values within a class, with the median shown by a central line. Lower-frequency ranges correspond to low-arousal or contact calls, while higher-frequency calls are associated with heightened arousal or distress, consistent with previous reports in bovine vocal studies.

**Figure 14:**
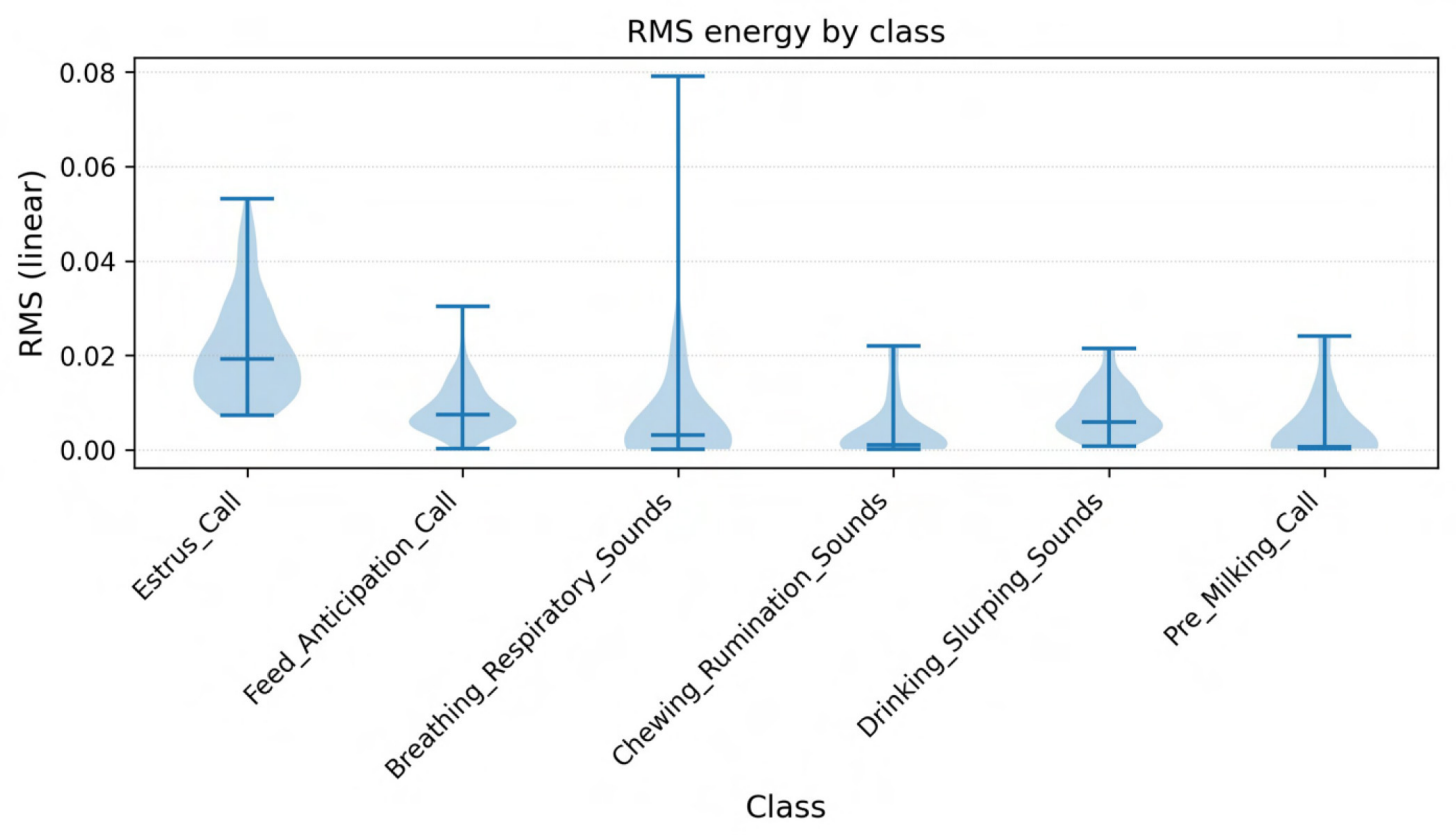
Violin plot of RMS energy by class. Comparison of average root-mean-square (RMS) energy among the top six vocalization classes. The RMS measure reflects overall signal intensity and vocal effort. Classes such as coughs and burps exhibit higher energy levels due to their abrupt, broadband nature, whereas harmonic moos and contact calls show lower, more stable energy envelopes.

To statistically evaluate these patterns, we applied non-parametric Kruskal–Wallis tests to four key features—clip duration, mean *F*_0_, spectral centroid, and RMS energy—across the twelve best-represented classes (*n*≥10: Breathing Respiratory Sounds, Chewing Rumination Sounds, Cough Calls, Drinking/Slurping Sounds, Estrus Call, Feed Anticipation Call, General Discomfort Call, Greeting Call, High Frequency Distress, Mating Excitement Call, Pain Related Call, and Pre-Milking Call). All four features showed highly significant differences among classes (duration: *H* = 143.6, *p* ≈ 3.0 × 10^−25^; mean *F*_0_: *H* = 220.9, *p* ≈ 3.4 × 10^−41^; spectral centroid: *H* = 255.5, *p* ≈ 2.0 × 10^−48^; RMS energy: *H* = 202.9, *p* ≈ 1.9 × 10^−37^), confirming that temporal, spectral, and energy-based parameters all carry strong class-discriminative information.

We then used Dunn's post-hoc tests with Bonferroni correction to identify which specific class pairs differed significantly. Across the 66 possible pairwise comparisons among the twelve classes, Dunn's tests identified 27, 25, 22, and 18 significant pairwise contrasts for duration, mean *F*_0_, spectral centroid, and RMS energy, respectively. For duration, Drinking/Slurping Sounds (median ≈ 30.0 s) were significantly longer than brief, high-arousal Mating Excitement Calls (median ≈ 4.3 s; $p_{\text{adj}}\approx 1.1\times 10^{-11}$) and Cough Calls (median ≈ 11.8 s; $p_{\text{adj}}\approx 2.7\times 10^{-8}$), illustrating how sustained drinking episodes contrast with short, impulsive vocalizations. For mean *F*_0_, low-frequency Breathing and Chewing Rumination Sounds (medians ≈ 118.5 Hz and ≈ 105.6 Hz) differed very strongly from high-frequency Estrus and Feed Anticipation Calls (medians ≈ 417.6 Hz and ≈ 415.8 Hz), with Bonferroni-corrected *p*-values effectively zero ($p_{\text{adj}}<10^{-12}$) and median differences exceeding 290–310 Hz. Spectral centroid exhibited similarly pronounced contrasts: non-vocal Breathing and Chewing Rumination Sounds (medians ≈ 2.4 kHz) had centroids more than 2 kHz higher than tonal Estrus and Feed Anticipation Calls (medians ≈ 370–395 Hz; *p*_adj_ ≈ 0), reflecting the broadband, noisy profile of respiratory and chewing events. Finally, RMS energy distinguished quiet contact-type calls from loud, high-arousal calls: Mating Excitement Calls (median ≈ −22.5 dB) were significantly more intense than Chewing Rumination Sounds and Pre-Milking Calls (medians ≈ −59.7 and ≈ −64.4 dB; $p_{\text{adj}}<10^{-10}$), indicating markedly higher vocal effort. Summary of Kruskal-Wallis statistics and the number of significant Dunn's post-hoc comparisons per feature are shown in Table 11.

**Table 11:** Kruskal–Wallis and Dunn's post-hoc results for four key acoustic features across the twelve best-represented behavioral classes.

**Feature**	***H* (Kruskal–Wallis)**	**df**	***p*-value**	**Significant Dunn pairs (Bonferroni)**
Duration (s)	143.61	11	2.99 × 10^−25^	27
Mean *F*_0_ (Hz)	220.86	11	3.42 × 10^−41^	25
Spectral centroid (Hz)	255.49	11	1.98 × 10^−48^	22
RMS energy (dB)	202.90	11	1.86 × 10^−37^	18

*H* and *p*-values refer to the Kruskal–Wallis omnibus test (df = 11). The final column reports the number of pairwise class contrasts that remained significant in Dunn's post-hoc tests after Bonferroni correction.

Feature distributions were visualized using violin plots (Figures 12–14), which highlight these class-specific signatures: Estrus and Feed Anticipation Calls cluster at higher F0 ranges and moderate spectral centroids; non-vocal respiratory and chewing sounds occupy a distinct region with very high spectral centroids and variable energy; and short, high-energy events such as coughs and distress calls show both elevated RMS and distinctive spectral profiles. Taken together, these Kruskal-Wallis and Dunn's test results show that all four key features vary strongly and systematically among behavioral classes, with mean F0 and spectral centroid providing particularly strong discrimination, and roughly one-third to two-fifths of all class pairs differing significantly for each feature even under stringent Bonferroni correction.

### 5.6 Linear mixed models and variance components

To quantify how much variation in acoustic features is attributable to vocalization class vs. recording context, we fitted linear mixed-effects models (LMMs) for four key features: clip duration, mean F0, spectral centroid, and RMS energy. For each feature, we used the same twelve best-represented behavioral classes as in the Kruskal-Wallis analysis and specified vocalization class as a fixed effect, with random intercepts for farm, barn zone, and microphone identity. This structure allowed us to partition variance into components associated with caller behavior (class), farm-level differences, local barn environment, microphone placement, and residual (within-class) variation.

Likelihood ratio tests comparing full models (including the fixed effect of class) against reduced models with only random effects showed that vocalization class significantly improved model fit for all four features. The likelihood ratio statistics were large and highly significant (duration: *LR* ≈ 177.4, df = 27, *p* ≈ 4.6 × 10^−24^; mean *F*_0_: *LR* ≈ 63.6, df = 27, *p* ≈ 8.7 × 10^−5^; spectral centroid: *LR* ≈ 789.8, df = 27, *p* ≈ 5.4 × 10^−149^; RMS energy: *LR* ≈ 332.0, df = 27, *p* ≈ 2.9 × 10^−54^), confirming that vocalization class explains a substantial amount of variation beyond farm, barn zone, and microphone effects. Full variance-component plots for all four linear mixed-effects models are provided in Supplementary Figures S3–S6.

Variance component estimates (Table 12) revealed that duration and spectral centroid are particularly structured by vocalization class and barn environment. For duration, vocalization class accounted for ~59.8% of the variance, with a further 38.4% attributable to barn zone and only 1.8% to residual variation. Spectral centroid showed a similar pattern, with 45.2% of variance explained by class and 29.4% by barn zone, compared with 5.5% by microphone and 19.9% residual. These results indicate that temporal and broad spectral-shape features carry strong class-specific signatures that remain robust after accounting for recording context, although local barn environment still contributes meaningfully.

**Table 12:** Proportion of variance in four key acoustic features explained by vocalization class, barn zone, microphone, and residual error in linear mixed-effects models.

**Feature**	**Vocalization class (%)**	**Barn zone (%)**	**Microphone (%)**	**Residual (%)**
Duration (s)	59.8	38.4	0.0	1.8
Mean *F*_0_ (Hz)	12.1	1.0	2.1	84.7
Spectral centroid (Hz)	45.2	29.4	5.5	19.9
RMS energy (dB)	46.7	0.5	10.3	42.5

Values are expressed as percentages of total variance.

In contrast, mean *F*_0_ exhibited a much larger residual component: only 12.1% of its variance was explained by vocalization class, with 1.0% from barn zone and 2.1% from microphone, while 84.7% remained as within-class residual variance. This suggests that *F*_0_ is influenced by substantial individual- or moment-level variation that is not captured by our current metadata, even though the class effect is still statistically significant. For RMS energy, vocalization class and recording hardware both played important roles: 46.7% of variance was attributed to class, 10.3% to microphone, and 42.5% to residual variation, underscoring that amplitude features are inherently sensitive to microphone placement and local noise, but still retain a strong behavioral signal. Overall, the LMM results corroborate the non-parametric tests by showing that key acoustic features contain substantial class-discriminative information, while explicitly quantifying the contributions of farm, barn environment, and microphone placement to the observed variability.

### 5.7 Principal component analysis of acoustic feature space

To visualize structure in the multivariate feature space, we applied principal component analysis (PCA) to the 24 standardized acoustic features extracted from all clips. The first principal component (PC1) explained 29.6% of the variance, the second (PC2) 16.0%, and the third (PC3) 14.1%, so that the first three PCs together captured 59.7% of the total variance. PCs 4 and 5 accounted for an additional 9.7% and 7.7%, respectively, with each subsequent component explaining <5% (scree plot in Supplementary Figure S7), indicating a clear elbow after the first 3–5 components.

Inspection of the PCA loadings showed that PC1 was dominated by spectral-shape and noisiness measures, with high positive loadings for spectral centroid, spectral bandwidth, spectral roll-off, and zero-crossing rate, as well as the first formant (*f*_1_). PC2 loaded most strongly on MFCC1, RMS energy mean and standard deviation, voiced ratio, and SNR, capturing an amplitude/voicing gradient. PC4 was primarily associated with *F*_0_ statistics (mean, minimum, and maximum), indicating that fundamental frequency variation is concentrated on a separate axis from broad spectral shape and energy.

The PC1–PC2 score distribution reveals a structured acoustic landscape: clips form broad clusters along a gradient from broadband, noisy events with high spectral centroid and zero-crossing rate (high PC1) to more tonal calls with concentrated low-frequency energy (low PC1), and along a second gradient from low-intensity, low-MFCC1 sounds to high-energy, strongly voiced events (high PC2). This multivariate view is consistent with the univariate and mixed-model analyses, confirming that temporal, spectral-shape, and energy features jointly organize cattle vocalizations in a low-dimensional space that can be exploited by downstream classifiers. Taken together, these dataset creation steps and multilevel statistical analyses provide a well-characterized acoustic benchmark for dairy cattle vocalizations, which we now interpret in the broader context of precision livestock welfare and existing bioacoustics literature in the Discussion.

## 6 Discussion

### 6.1 Significance for AI and big data

The dataset presented in this study contributes directly to advancing AI and data-driven approaches in animal bioacoustics. Unlike prior collections that were limited in size or scope, this corpus offers both scale and diversity, providing 569 annotated clips (expanded to ~ 2,900 after augmentation for modeling) across 48 behavioral classes. Such breadth is crucial for machine learning applications, where model performance depends on exposure to both common and rare events. By incorporating high-arousal calls (e.g., estrus, distress), affiliative calls (e.g., contact, maternal), and non-vocal sounds (e.g., breathing, burps), the dataset enables algorithms to learn the acoustic signatures of a wide behavioral spectrum rather than a narrow subset of conditions.

Equally important is the dataset's ecological realism. Machine learning models trained on clean, noise-free recordings often fail when deployed in real barn environments. By retaining authentic background noise and overlapping acoustic events, this resource ensures that computational models developed from it are robust to the challenges of deployment. The multimicrophone and multimodal setup adds further depth, allowing researchers to explore cross-validation across equipment types and zones, or to align acoustic features with behavioral context captured in video. This design makes the dataset valuable not only for supervised classification but also for self-supervised learning, representation learning, and transfer learning, areas where large, heterogeneous datasets are particularly impactful. The Kruskal-Wallis, Dunn's post-hoc tests, and linear mixed-effects models together show that key acoustic features (duration, F0, spectral centroid, RMS) vary systematically between behavioral classes and are not dominated by farm, barn zone, or microphone artifacts. Variance components indicate that class explains a substantial proportion of the variability, with environmental and hardware effects contributing smaller, secondary components.

Finally, the integration of FAIR-compliant metadata and standardized acoustic features (Praat, librosa, openSMILE) ensures that the dataset is interoperable with wider AI research ecosystems. Researchers can directly apply established pipelines for feature selection, dimensionality reduction, or deep learning input preparation, reducing barriers to reproducibility. Because the pipeline relies on generic time-frequency features and FAIR metadata, the same framework can be extended to other livestock species (e.g., sheep, goats, pigs) and integrated into cross-species AI models for farm animal welfare. In this way, the dataset bridges the gap between animal behavior research and contemporary AI methodologies, situating bovine vocalization analysis firmly within the domain of big data science.

### 6.2 Limitations and challenges

While comprehensive, the dataset is not without limitations. The most prominent challenge lies in class imbalance, with a small number of categories such as estrus and feeding anticipation dominating the corpus, while rare events like drinking competition calls or novel environment response calls are represented by only a few clips. This long-tailed distribution mirrors behavioral ecology but complicates model training, as classifiers may overfit frequent categories while neglecting rare yet ethologically significant ones. Augmentation strategies can mitigate this bias, but they cannot fully replace naturally occurring data.

Another limitation arises from the subjectivity of manual annotation. Although rigorous protocols and cross-validation by multiple annotators were employed, certain call types are inherently ambiguous, especially when overlapping with background noise or occurring in complex social contexts. Labels such as “frustration” or “distress” are based on behavioral inference, which, while grounded in ethology, cannot capture internal emotional states with absolute certainty. This highlights the need for future datasets to integrate multimodal physiological or sensor-based validation to strengthen label reliability. Manual segmentation and annotation, while ensuring high fidelity, remain time-intensive and may limit scalability without semi-automated approaches. Future work integrating automated detectors or active learning frameworks could alleviate this bottleneck while preserving annotation accuracy.

The acoustic environment itself also posed challenges. Commercial barns are characterized by persistent mechanical noise and overlapping vocal activity, which, although essential for ecological realism, reduce signal clarity. Even after careful denoising and filtering, residual interference remains in some clips. While this realism enhances deployment value, it also increases computational demands for segmentation, feature extraction, and classification. Researchers applying advanced models to this dataset should therefore be aware that noise robustness remains an open problem.

A key limitation is that we did not estimate source-microphone distance for each clip. Microphones were placed in ecologically realistic positions within barn zones, but per-clip distance and propagation paths were not measured. As a result, amplitude-related features combine caller effort, distance, and local acoustics; future work using video tracking or localization technology could explicitly model distance and better separate source-level and environmental effects.

Similarly, cow-level factors such as age, parity, and lactation stage were not fully encoded in the public metadata, and we did not perform systematic parity- or age-stratified analyses. Our results therefore reflect aggregate patterns across mixed groups of heifers and multiparous cows. Future studies should incorporate standardized animal-level descriptors to disentangle behavioral, physiological, and life-history effects on vocalization structure.

Finally, although the dataset is large by livestock bioacoustics standards, it remains modest compared to big data benchmarks in other AI fields such as speech recognition or computer vision. Expanding the temporal coverage (e.g., across seasons, farms, and breeds) and enlarging the sample size would further strengthen its generalisability and enable the training of more complex deep learning architectures.

### 6.3 Outlook and future directions

The current dataset establishes a foundation, but it also opens several avenues for expansion and methodological innovation. One promising direction is the incorporation of longitudinal recordings that capture vocal behavior across different seasons, management practices, and life stages. Extending coverage beyond three farms and including diverse breeds would improve representativeness and allow for comparative studies across genetic and environmental contexts.

Another opportunity lies in multimodal integration. While this dataset already links audio to video and manual annotations, future work could align vocal data with physiological markers (e.g., heart rate, cortisol levels, rumination sensors) to provide multi-layered evidence of welfare states. Such integration would reduce ambiguity in behavioral labels and strengthen the interpretability of acoustic indicators.

From a computational perspective, the dataset is particularly well-suited for exploring emerging AI paradigms. Large, heterogeneous acoustic corpora are valuable for training self-supervised models that learn general-purpose representations before fine-tuning for specific tasks such as estrus detection, welfare monitoring, or individual identification. Similarly, transfer learning from bovine vocalizations to related species–or from human speech models to livestock contexts–presents opportunities for cross-domain innovation.

Finally, the dataset highlights the importance of open, standardized resources in agricultural AI. By adhering to FAIR principles, this work contributes to a growing movement toward reproducible, community-driven datasets in animal science. Establishing shared benchmarks for livestock bioacoustics, similar to ImageNet or LibriSpeech in computer vision and speech research, would accelerate progress by enabling systematic comparisons of models and fostering collaborative development. The resource introduced here can serve as an early step in that direction, encouraging both the scaling of future datasets and the refinement of analytical tools tailored to animal vocal behavior. These considerations set the stage for our conclusion, where we summarize the dataset's contributions and its potential role in advancing digital agriculture and big data applications in animal science.

## 7 Conclusion

In this work, we introduced one of the most comprehensive bovine vocalization datasets assembled to date, integrating 569 curated original clips across 48 behavioral classes recorded in authentic barn environments. By combining multi-microphone audio capture, complementary video observations, and detailed ethology-driven annotations, the dataset provides an ecologically valid resource for advancing both animal behavior research and computational modeling. Preprocessing steps ensured that clips were denoised, segmented, and paired with rich metadata, while feature extraction pipelines generated interpretable acoustic descriptors aligned with welfare science.

The dataset makes three key contributions. First, it broadens the empirical foundation for studying cattle vocal behavior beyond the constraints of controlled laboratory recordings, embracing the acoustic complexity of real farm settings. Second, it provides a reproducible, FAIR-compliant framework with transparent metadata and feature definitions, positioning bovine bioacoustics within the wider ecosystem of big data research. Third, it offers a benchmark corpus for the development and testing of AI methods, from supervised classification to emerging approaches such as self-supervised representation learning.

Taken together, these contributions establish a foundation for non-invasive, data-driven approaches to animal welfare monitoring and precision livestock management. While challenges remain in scaling, class balance, and multimodal integration, this dataset represents a critical step toward creating the robust, reproducible resources needed to give cattle a “digital voice” in future smart farming systems.

## Data Availability

The curated audio dataset and associated metadata are deposited in Zenodo under restricted access (DOI: 10.5281/zenodo.17764250), with access provided through a Data Access Agreement to protect farm confidentiality. Metadata, documentation, and code are publicly available, and the open-source preprocessing and feature extraction pipeline is available at https://github.com/mooanalytica/bovine-bioacoustics-preprocessing.
