# Supplementary material for: Big data approaches to bovine bioacoustics: a FAIR-compliant dataset and scalable ML framework for precision livestock welfare
Source: Front Big Data. 2026 Jan 16;8:1723155. doi: 10.3389/fdata.2025.1723155 (PMC12855049; doi:10.3389/fdata.2025.1723155)
Supplement: Supplementary file 1 [file Table_1.docx]

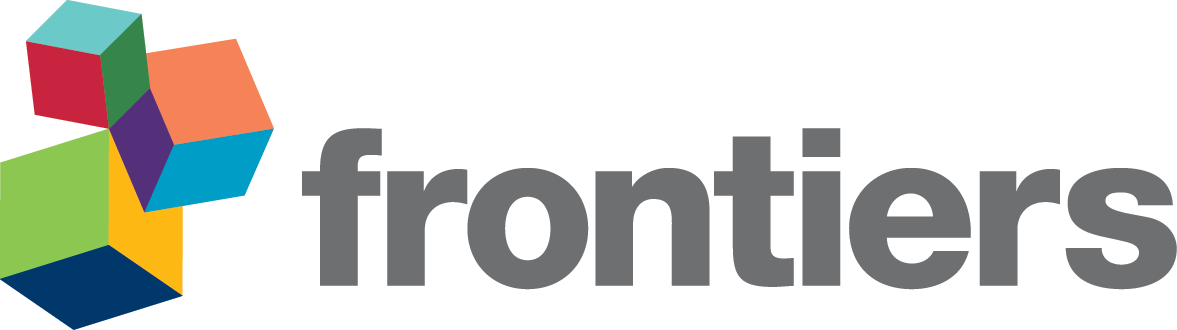


# *Supplementary Material*

## 1 SUPPLEMENTARY TABLES AND FIGURES

**Table S1.** Composition of the curated bovine vocalization dataset by main category and subcategory. For each subcategory, we report the number of annotated clips (*N*_clips_), total and median clip duration (seconds), minimum and maximum duration, number of farms, barn zones, and microphones represented, and mean signal-to-noise ratio (Mean SNR, dB).

| Main category | Subcategory | *N*clips | Total duration (s) | Median duration (s) | Min duration (s) | Max duration (s) | *N*farms | *N*barn zones | *N*microphones | Mean SNR (dB) |
| --- | --- | --- | --- | --- | --- | --- | --- | --- | --- | --- |
| Distress & Pain | General Discomfort Call | 11 | 207.82 | 18.10 | 10.26 | 38.16 | 3 | 4 | 3 | 22.81 |
| Distress & Pain | High Frequency Distress | 10 | 196.13 | 18.05 | 14.00 | 33.76 | 2 | 3 | 3 | 21.73 |
| Distress & Pain | Illness Indication Call | 7 | 82.87 | 11.35 | 10.59 | 14.79 | 3 | 3 | 2 | 30.01 |
| Distress & Pain | Injury Response Call | 1 | 13.71 | 13.71 | 13.71 | 13.71 | 1 | 1 | 1 | 17.11 |
| Distress & Pain | Pain Related Call | 11 | 194.89 | 15.51 | 10.44 | 30.70 | 3 | 4 | 3 | 25.26 |
| Environmental & Situational | Novel Environment Call | 2 | 65.89 | 32.94 | 32.19 | 33.70 | 1 | 2 | 2 |  |
| Estrus & Mating Behavior | Estrus Call | 117 | 1587.10 | 13.44 | 13.07 | 15.02 | 3 | 3 | 3 |  |
| Estrus & Mating Behavior | Mating Excitement Call | 10 | 44.31 | 4.32 | 3.64 | 5.49 | 1 | 1 | 1 |  |
| Feeding & Hunger Related | Empty Feeder Call | 6 | 144.57 | 21.89 | 15.98 | 34.22 | 3 | 2 | 2 | 16.80 |
| Feeding & Hunger Related | Feed Anticipation Call | 112 | 1579.54 | 13.07 | 11.44 | 35.77 | 2 | 2 | 3 | 18.65 |
| Feeding & Hunger Related | Feed Competition Call | 3 | 68.05 | 17.85 | 16.11 | 34.09 | 1 | 1 | 1 | 17.89 |
| Feeding & Hunger Related | Feed Quality Response | 5 | 84.92 | 17.92 | 9.89 | 24.20 | 2 | 2 | 2 | 14.54 |
| Feeding & Hunger Related | Hunger Frustration Call | 6 | 252.21 | 43.66 | 27.43 | 56.65 | 2 | 2 | 3 | 23.65 |
| Feeding & Hunger Related | Post Feeding Satisfaction | 9 | 195.54 | 24.30 | 11.85 | 33.35 | 1 | 1 | 1 | 11.94 |
| Maternal & Calf Communication | Calf Contact Call | 6 | 346.92 | 57.83 | 18.59 | 97.00 | 2 | 2 | 2 |  |
| Maternal & Calf Communication | Maternal Response Call | 1 | 321.04 | 321.04 | 321.04 | 321.04 | 1 | 1 | 1 |  |
| Maternal & Calf Communication | Mother Separation Call | 9 | 124.79 | 13.00 | 11.10 | 24.29 | 2 | 2 | 1 |  |
| Milking & Handling | Handling Stress Call | 3 | 73.17 | 24.90 | 23.26 | 25.01 | 1 | 1 | 1 |  |
| Milking & Handling | Milking Discomfort Call | 9 | 231.56 | 24.72 | 20.93 | 35.38 | 2 | 1 | 2 |  |
| Milking & Handling | Pre Milking Call | 12 | 273.19 | 21.73 | 20.21 | 30.14 | 3 | 1 | 3 |  |
| Milking & Handling | Restraint Protest Call | 1 | 20.88 | 20.88 | 20.88 | 20.88 | 1 | 1 | 1 |  |
| Non-Vocal Sounds | Breathing Respiratory Sounds | 77 | 1347.37 | 15.27 | 4.47 | 50.22 | 3 | 3 | 3 | 26.47 |
| Non-Vocal Sounds | Chewing Rumination Sounds | 31 | 1225.60 | 24.75 | 2.80 | 444.90 | 3 | 3 | 4 | 16.94 |
| Non-Vocal Sounds | Cough Calls | 11 | 134.37 | 11.77 | 10.84 | 18.73 | 2 | 1 | 1 |  |
| Non-Vocal Sounds | Drinking Slurping Sounds | 17 | 620.40 | 30.02 | 12.48 | 88.52 | 2 | 3 | 3 | 71.48 |
| Non-Vocal Sounds | Licking Sounds | 3 | 104.42 | 20.02 | 19.46 | 64.95 | 2 | 2 | 1 | 54.85 |
| Non-Vocal Sounds | Movement Associated Sounds | 3 | 52.35 | 15.24 | 11.90 | 25.20 | 2 | 3 | 2 | 66.86 |
| Social Recognition & Interaction | Greeting Call | 11 | 162.17 | 12.38 | 11.08 | 26.68 | 3 | 3 | 3 | 42.38 |
| Social Recognition & Interaction | Group Contact Call | 6 | 499.57 | 76.89 | 13.75 | 169.70 | 3 | 4 | 3 |  |
| Social Recognition & Interaction | Herd Coordination Call | 7 | 395.60 | 12.69 | 10.32 | 266.99 | 4 | 3 | 3 | 19.81 |
| Social Recognition & Interaction | Individual Recognition Call | 7 | 85.03 | 12.38 | 10.06 | 13.36 | 2 | 3 | 3 | 67.79 |
| Social Recognition & Interaction | Proximity Maintenance Call | 1 | 12.19 | 12.19 | 12.19 | 12.19 | 1 | 1 | 1 |  |
| Social Recognition & Interaction | Response Exchange Call | 7 | 123.13 | 18.07 | 13.36 | 23.13 | 3 | 4 | 3 | 44.02 |
| Social Recognition & Interaction | Social Bonding Call | 9 | 481.17 | 58.32 | 17.90 | 121.56 | 3 | 5 | 4 | 75.61 |
| Water & Thirst Related | Dehydration Distress Call | 5 | 73.50 | 13.26 | 10.21 | 20.89 | 3 | 2 | 2 | 59.45 |
| Water & Thirst Related | Drinking Competition Call | 2 | 65.82 | 32.91 | 18.02 | 47.81 | 1 | 1 | 1 | 72.47 |
| Water & Thirst Related | Thirst Anticipation Call | 9 | 163.38 | 14.25 | 10.07 | 28.07 | 3 | 2 | 2 | 56.96 |
| Water & Thirst Related | Water Frustration Call | 8 | 236.46 | 28.08 | 10.31 | 57.81 | 2 | 2 | 2 | 78.24 |
| Water & Thirst Related | Water Quality Response | 4 | 78.88 | 20.14 | 10.60 | 28.02 | 2 | 2 | 2 | 65.67 |


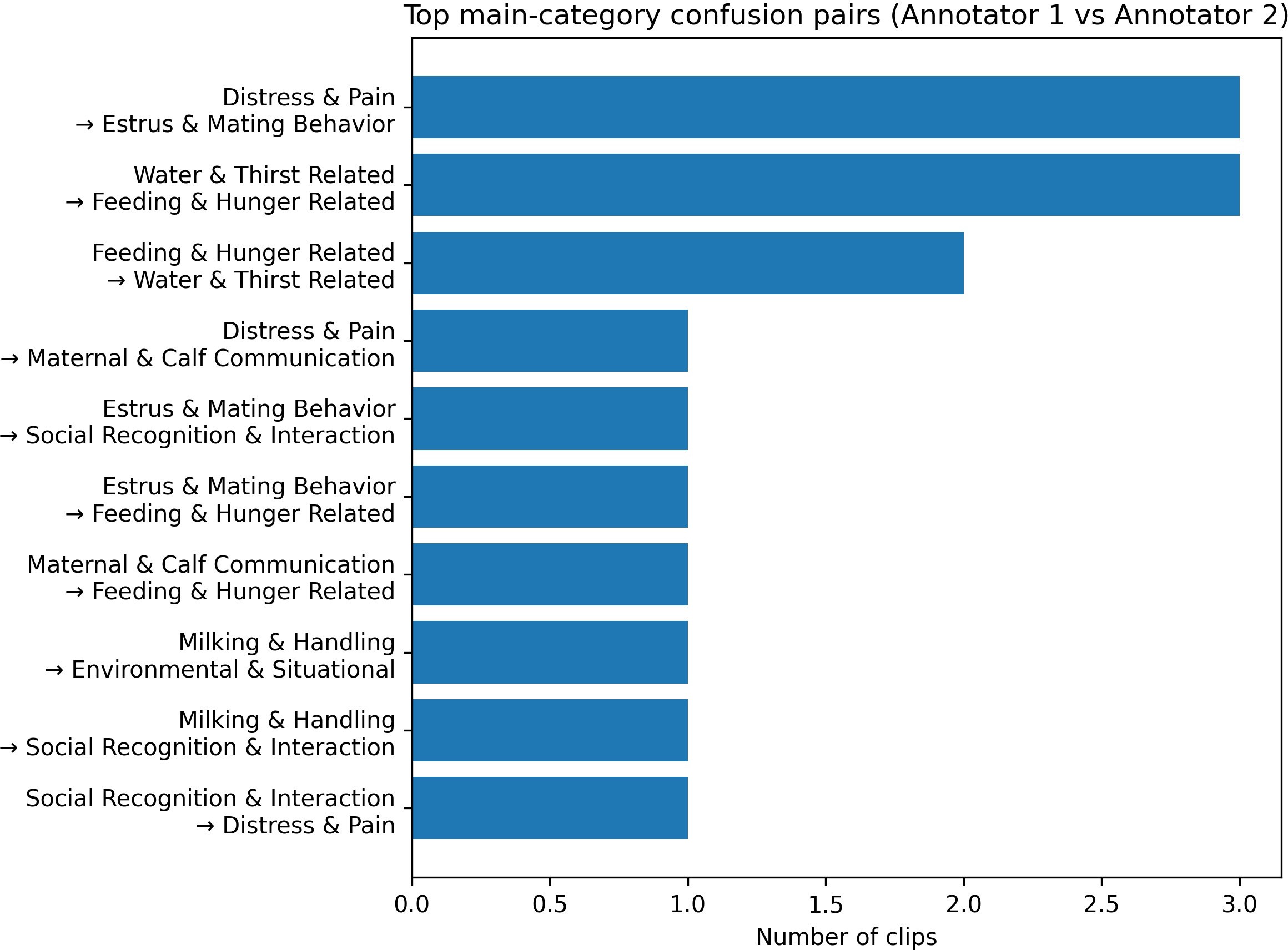


**Figure S1. Inter-annotator agreement - Main-category confusion pairs between annotators.** Top main-category mismatches between Annotator 1 and Annotator 2 in the 150-clip subset, showing that disagreements are rare and mainly occur between conceptually adjacent categories.


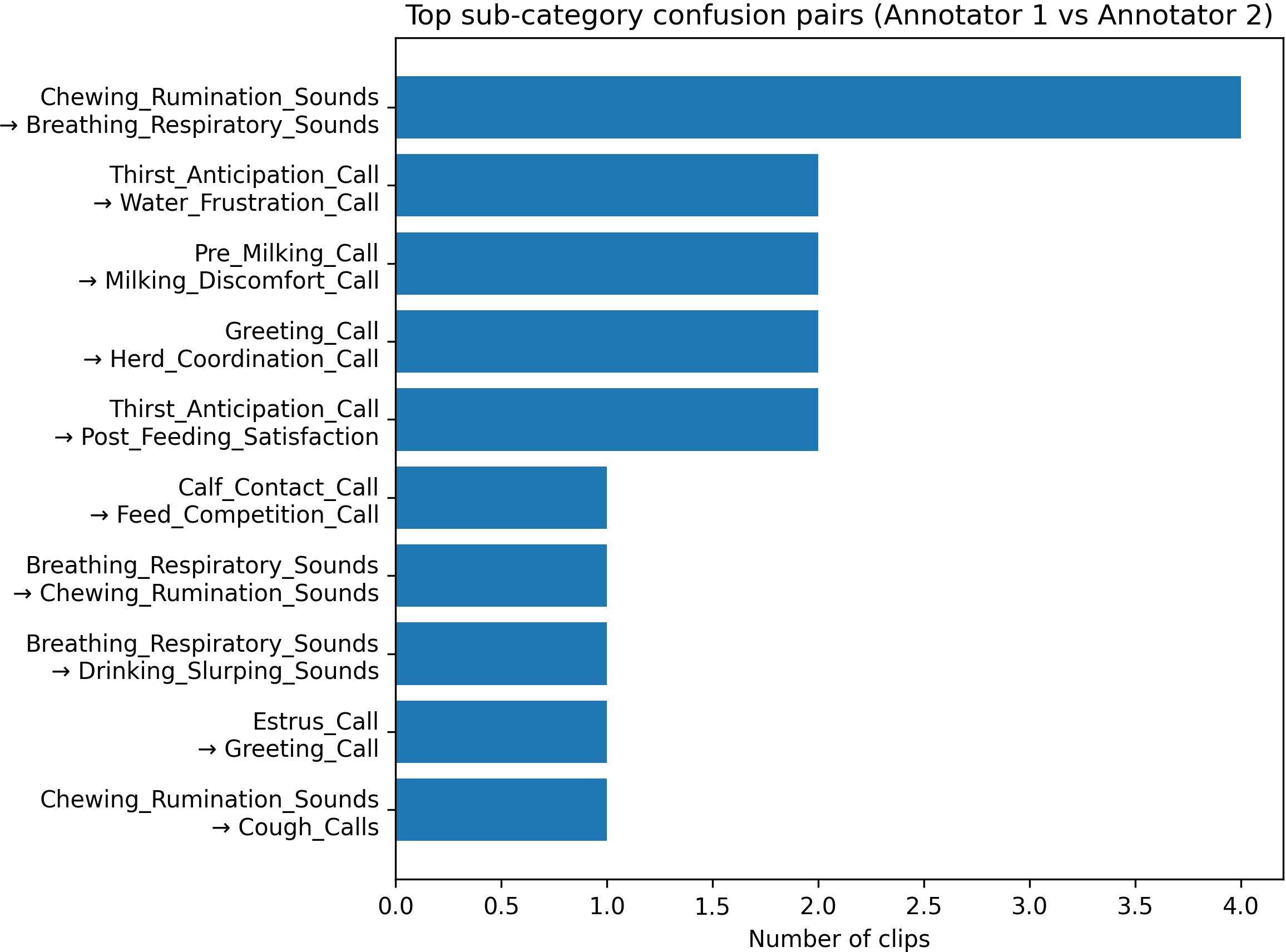


**Figure S2. Inter-annotator agreement - Sub-category confusion pairs within the same main category.** Top sub-category mismatches for clips where annotators agreed on the main category, illustrating that disagreements cluster in a few acoustically and contextually similar call types rather than being broadly distributed across all sub-categories.


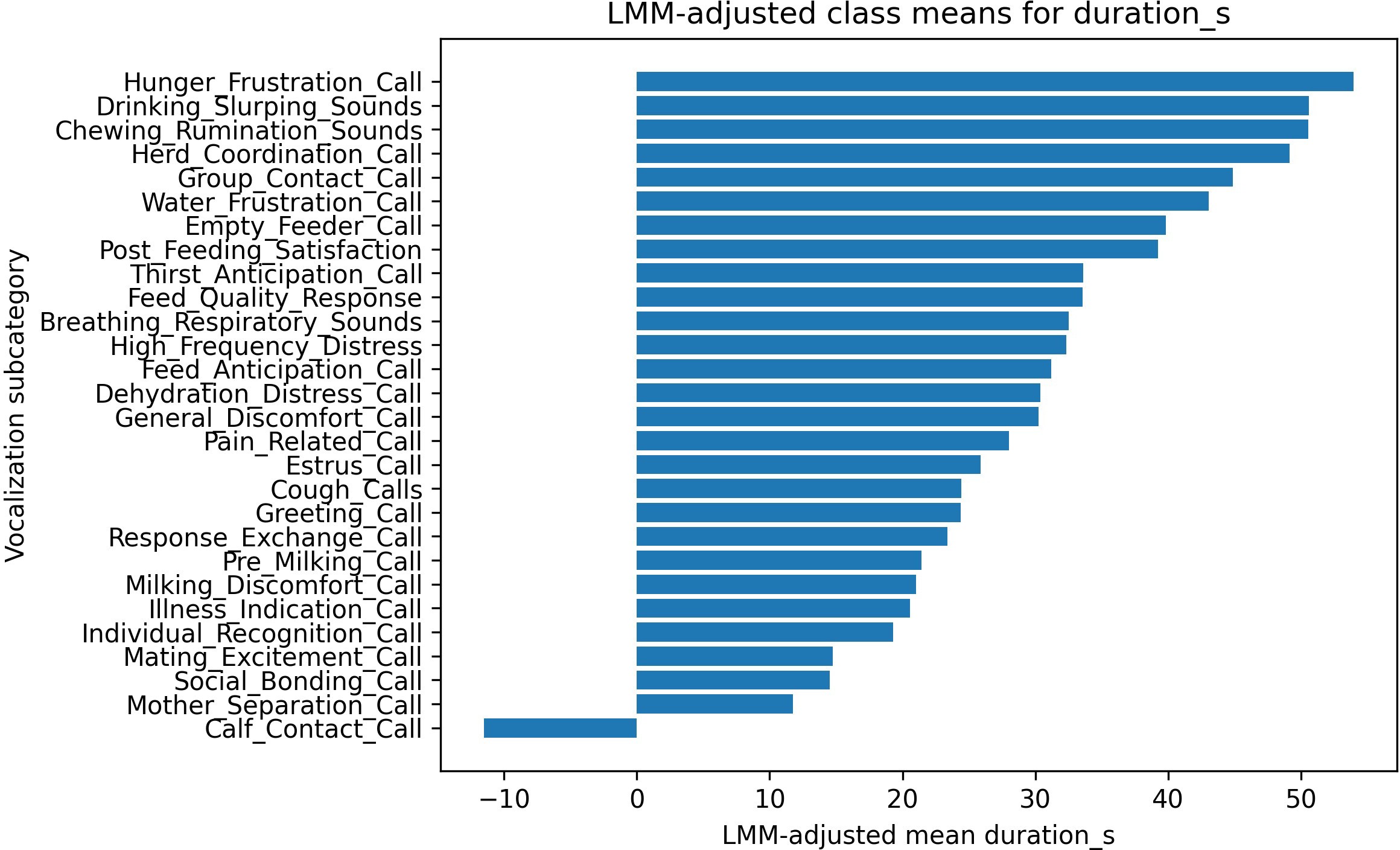


**Figure S3.** Linear mixed-effects model variance components for clip duration, showing the proportion of variance attributed to vocalization class, barn zone, microphone, and residual error.


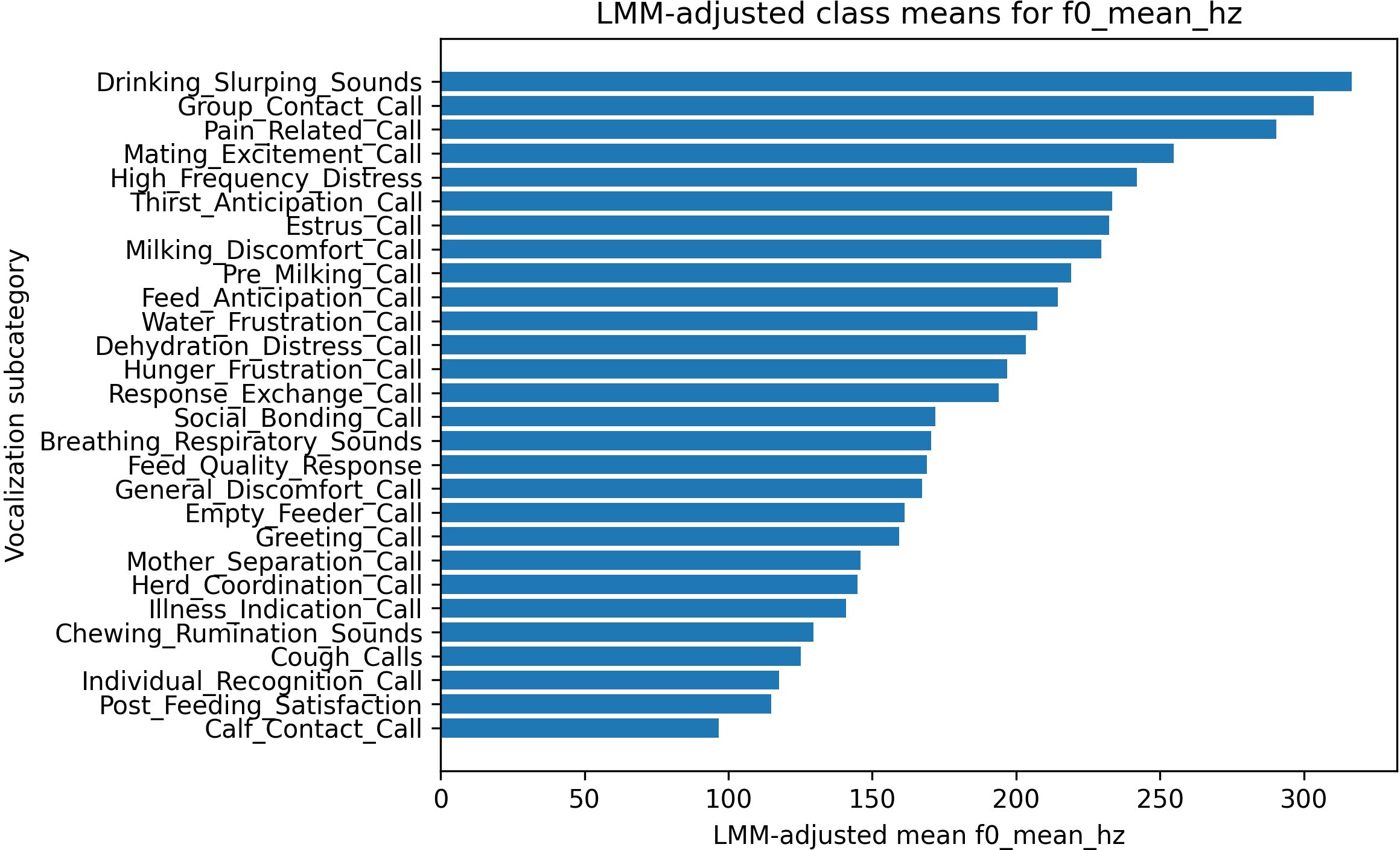


**Figure S4.** Linear mixed-effects model variance components for mean *F*_0_, highlighting the relatively large residual variance compared with effects of class, barn zone, and microphone.


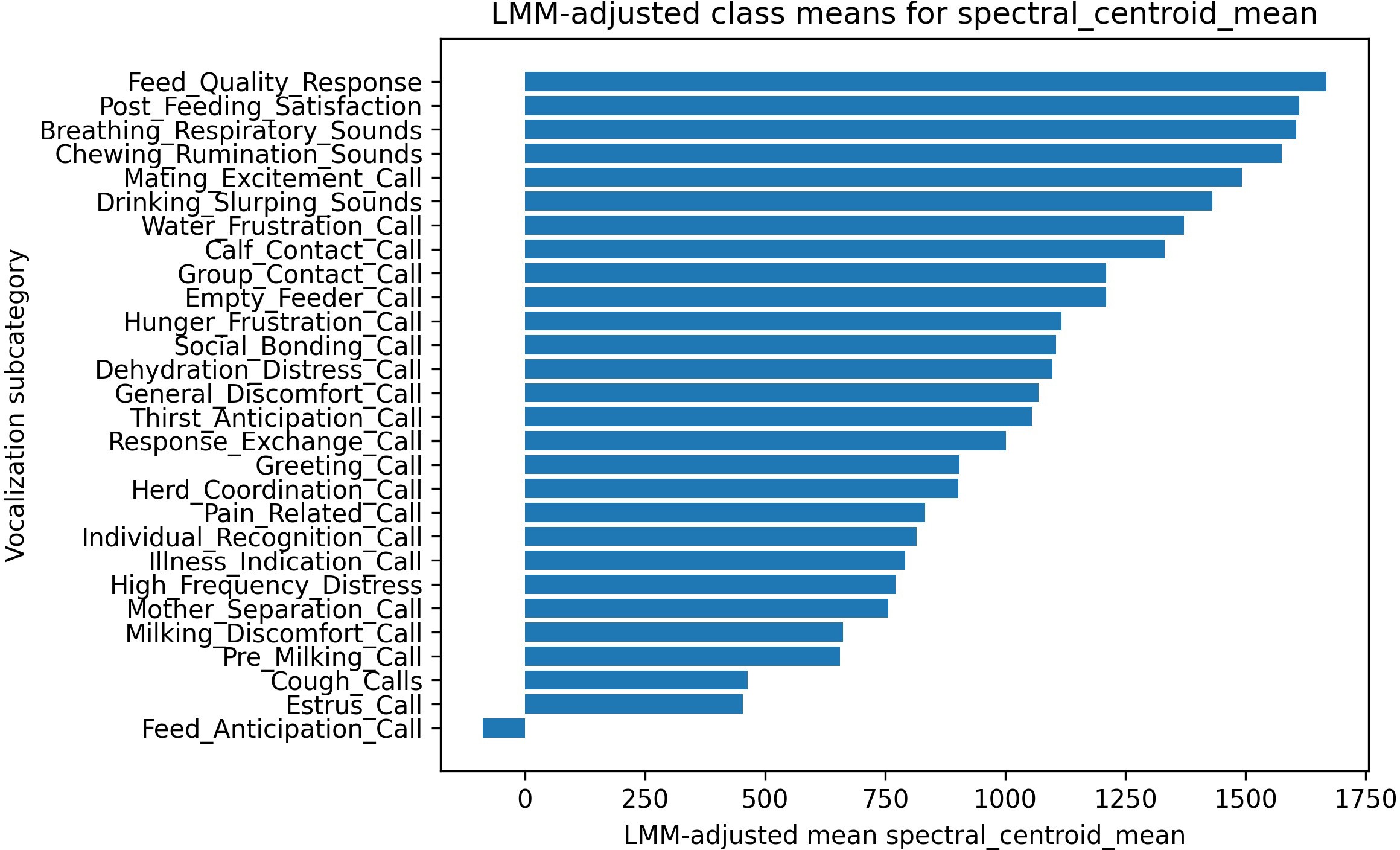


**Figure S5.** Linear mixed-effects model variance components for spectral centroid, indicating strong structuring by vocalization class and barn zone, with smaller contributions from microphone and residual error.


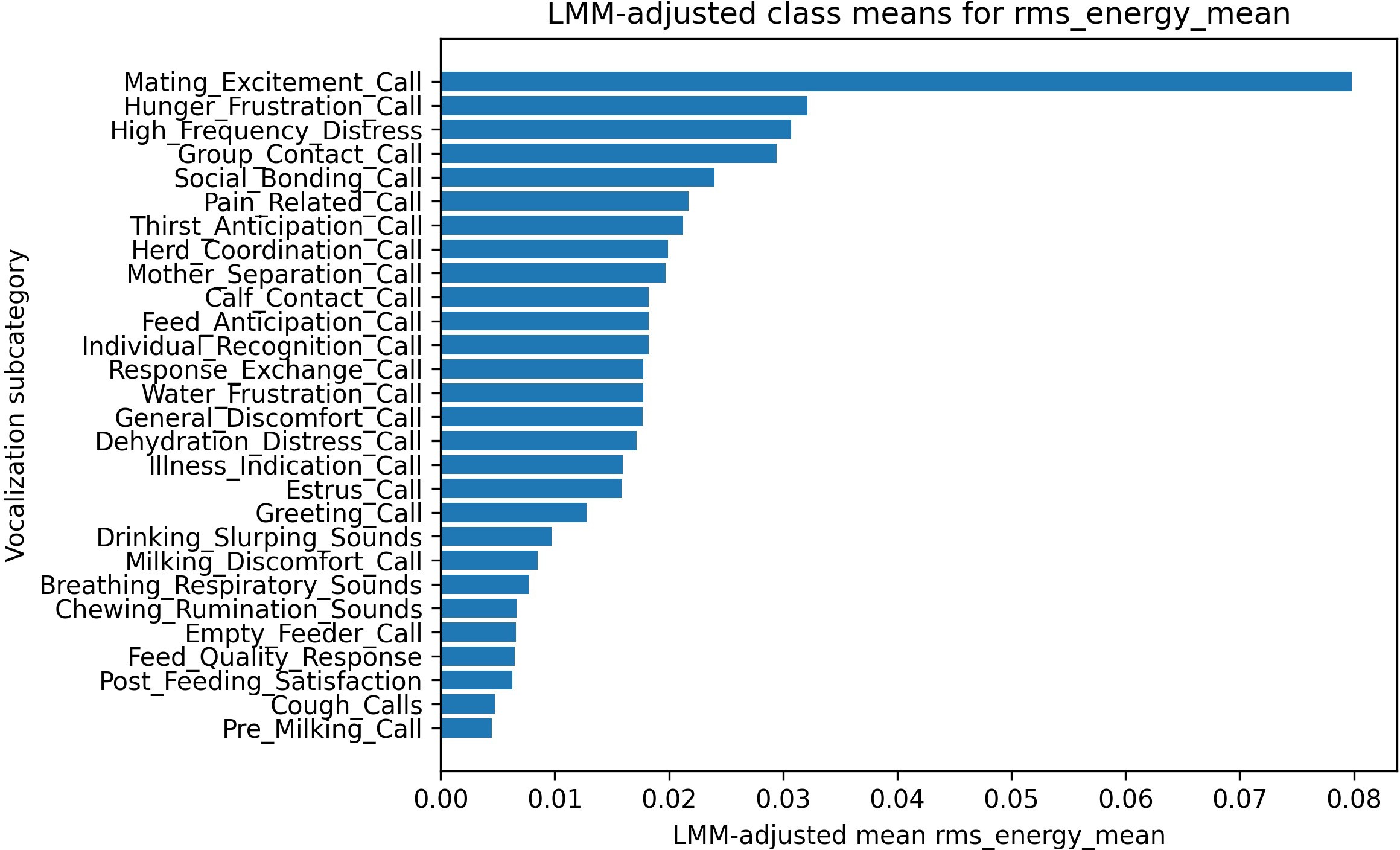


**Figure S6.** Linear mixed-effects model variance components for RMS energy, showing substantial effects of vocalization class and microphone, alongside notable residual variability.


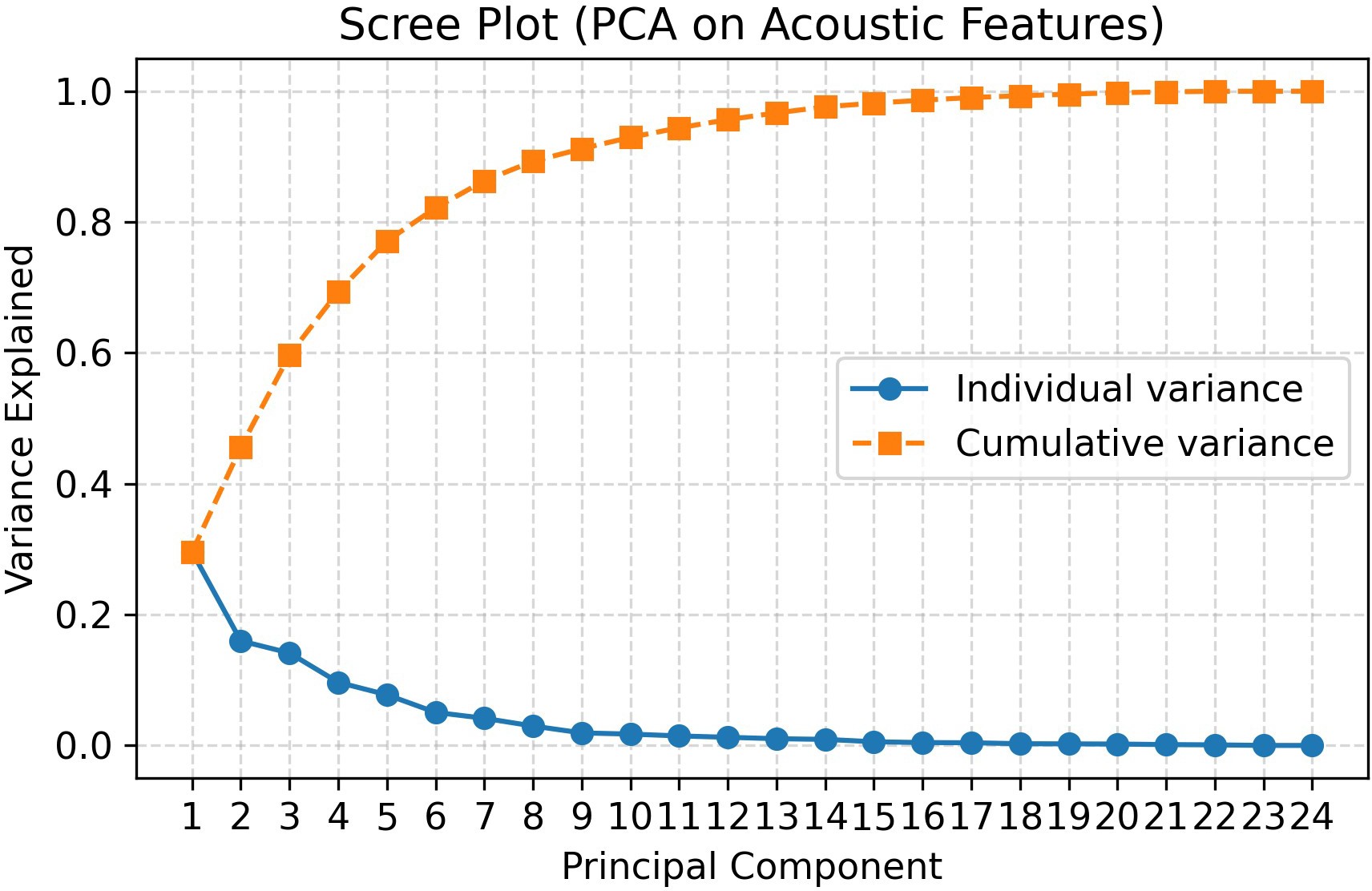


**Figure S7.** Scree plot showing variance explained by each principal component (blue) and cumulative variance (orange) for the 24-feature acoustic space.
